# Non-clinical and Pre-clinical Testing to Demonstrate Safety of the Barostim Neo Electrode for Activation of Carotid Baroreceptors in Chronic Human Implants

**DOI:** 10.3389/fnins.2017.00438

**Published:** 2017-08-02

**Authors:** Seth J. Wilks, Seth A. Hara, Erika K. Ross, Evan N. Nicolai, Paul A. Pignato, Adam W. Cates, Kip A. Ludwig

**Affiliations:** ^1^CVRx, Inc. Minneapolis, MN, United States; ^2^Department of Neurologic Surgery, Mayo Clinic Rochester, MN, United States; ^3^AtriCure, Inc. Mason, OH, United States

**Keywords:** baroreflex activation therapy, electrode characterization, neuromodulation, bioelectronic medicine, electroceutical, preclinical, heart failure, hypertension

## Abstract

The Barostim neo™ electrode was developed by CVRx, Inc.to deliver baroreflex activation therapy (BAT)™ to treat hypertension and heart failure. The neo electrode concept was designed to deliver electrical stimulation to the baroreceptors within the carotid sinus bulb, while minimizing invasiveness of the implant procedure. This device is currently CE marked in Europe, and in a Pivotal (akin to Phase III) Trial in the United States. Here we present the *in vitro* and *in vivo* safety testing that was completed in order to obtain necessary regulatory approval prior to conducting human studies in Europe, as well as an FDA Investigational Device Exemption (IDE) to conduct a Pivotal Trial in the United States. Stimulated electrodes (10 mA, 500 μs, 100 Hz) were compared to unstimulated electrodes using optical microscopy and several electrochemical techniques over the course of 27 weeks. Electrode dissolution was evaluated by analyzing trace metal content of solutions in which electrodes were stimulated. Lastly, safety testing under Good Laboratory Practice guidelines was conducted in an ovine animal model over a 12 and 24 week time period, with results processed and evaluated by an independent histopathologist. Long-term stimulation testing indicated that the neo electrode with a sputtered iridium oxide coating can be stimulated at maximal levels for the lifetime of the implant without clinically significant dissolution of platinum or iridium, and without increasing the potential at the electrode interface to cause hydrolysis or significant tissue damage. Histological examination of tissue that was adjacent to the neo electrodes indicated no clinically significant signs of increased inflammation and no arterial stenosis as a result of 6 months of continuous stimulation. The work presented here involved rigorous characterization and evaluation testing of the neo electrode, which was used to support its safety for chronic implantation. The testing strategies discussed provide a starting point and proven framework for testing new neuromodulation electrode concepts to support regulatory approval for clinical studies.

## Introduction

Implantable electrodes that are intended to electrically activate the nervous system for therapeutic purposes—commonly referred to as neuromodulation devices, bioelectronic medicines, or electroceuticals (Famm et al., 2013; Birmingham et al., 2014; Reardon, 2014; Olofsson and Tracey, 2017)–are estimated to become between a 6 and 10 billion dollar industry by 2020 (2015; 2016). Unfortunately, the non-clinical (bench) and pre-clinical (animal) safety testing strategy for implantable electrode designs to obtain regulatory approval to conduct human studies is often difficult for academics and small businesses navigating the regulatory pathway for the first time to divine. Although there are some excellent references from the FDA, such as the “Investigational Device Exemption (IDE) Guidance for Retinal Prostheses,” these references provide general outlines of the factors that should be considered in developing tests, and lack the detail necessary to formulate an actionable safety testing strategy. The FDA routinely provides project-specific feedback under the FDA's Pre-Submission program, but this process typically requires the sponsor to first submit a detailed testing strategy to the FDA for comment. Consequently, a detailed example of a testing strategy to ensure electrical stimulation safety that has led to an FDA IDE and/or approval to conduct human studies through a European notified body would provide a critical frame of reference for the Pre-Submission process.

In this manuscript, we describe the stimulation safety testing strategy successfully used to receive CE mark and FDA IDE approval for the second generation baroreflex activation therapy (BAT)™ system. Previous studies have demonstrated the efficacy of BAT as a supplement to pharmacological therapies for treatment resistant hypertension and heart failure. To accomplish this, BAT electrically activates the baroreceptors on the carotid sinus, thereby resulting in the transmission of afferent signals to the brain that are then interpreted as an increase in blood pressure. In response, sympathetic inhibition and heart rate decrease is initiated through the body's own control mechanisms, resulting in a decrease in blood pressure. Although this effect has been known since 1967, technological limitations at the time prevented further development of BAT as a safe and effective therapy (Heusser et al., 2010; Zhang et al., 2014; Victor, 2015). In recent years device manufacturer CVRx® received a CE mark for hypertension and received a Humanitarian Device Exemption (HDE) from the FDA for their first-generation more invasive BAT system on the basis of positive data from trials conducted in both Europe and the United States (Scheffers et al., 2010; Bisognano et al., 2011; FDA, 2014b). The second-generation minimally-invasive system, neo, has demonstrated similar efficacy to the first-generation system in open-label clinical studies with a greatly improved safety profile (Hoppe et al., 2012; Abraham et al., 2015).

### Background and rationale for Barostim neo testing methods for regulatory approval

A common misconception is that stimulation safety testing for regulatory approval provides conclusive evidence that unwanted electrochemical reactions do not occur, or lead to electrode dissolution and/or potential toxicity to tissue near the electrode. It is also a misconception that safety testing guarantees that stimulation levels never exceed levels that would drive local neural activity beyond physiological norms leading to an “excitotoxic” change in neural function or neural death. For example, although stimulation through platinum/iridium (Pt/Ir) alloy electrodes at charge densities over 20 μC/cm^2^ is known to cause platinum dissolution *in vivo* (Robblee et al., 1983), existing spinal cord stimulators are approved for charge densities as high as 150 μC/cm^2^ (FDA, 2014a). Post-mortem studies of cochlear implants have shown platinum particulate matter in the vicinity of the implanted electrode that has generated a particulate-specific foreign body response (Clark et al., 2013, 2014; Nadol et al., 2014; Spiers et al., 2016). However, these stimulation-induced physiological responses have not been directly linked to any clinically significant adverse effects in the patient, especially in context of the much larger initial and chronic insult to tissue caused by the implant itself. There are only data suggesting that extensive foreign body reaction to the implant could cause a decrease in clinical utility of the implant, therefore subjecting the patients to the surgical risk of the implant without providing as much benefit (Nadol et al., 2014).

Consequently, the primary goal of non-clinical testing of stimulation safety for regulatory approval is to better define the risks introduced by stimulation so that an accurate assessment of likely benefits vs. risks to the patient can be made. The thoroughness of such risk-benefit assessment must be balanced by the burden of the testing plan to the manufacturer. Animal cohort size and study duration must be large enough to reasonably assess a risk-benefit profile, but not too large to introduce additional burden on the manufacturer, which would be prohibitive. The cost of the individual tests performed is often insignificant in comparison to the time it takes to perform the tests. For example, a small neuromodulation company may pay well in excess of 10 million dollars a year for employee salaries, office space, etc., meaning that adding months to the timeline for a test on the critical path to approval adds significant financial burden to the company as the test is performed. This is an important consideration, as the FDA is bound by the “Least Burdensome” Provision of the FDA Modernization Act of 1997, which is intended to streamline the regulatory process and eliminate unnecessary burdens that may delay the marketing of beneficial new products (FDA, 2002).

The Barostim neo is intended for patients with treatment resistant hypertension or New York Heart Association functional class III heart failure with ejection fraction ≤35% on stable guideline-directed medical therapy (Scheffers et al., 2010; Abraham et al., 2015). The 3 year mortality rate for patients with functional class III heart failure with reduced ejection fraction is 30% (MAGGIC, 2012), and population studies indicate that the risks of myocardial infarction, stroke, heart failure, and renal failure directly relate to blood pressure levels (Sarafidis and Bakris, 2008). Given the severity of these conditions and short-term prognoses, there is a degree of tolerance for potential risks that are introduced by an electrically active implant, if proven beneficial. As a result, the goals of non-clinical stimulation safety testing for regulatory approval were to evaluate (1) the likelihood the implant would fail to provide therapeutic efficacy prior to 10 years^1^ due to stimulation-induced dissolution of the electrode or damage to local nervous tissue and (2) clinically-significant damage to local nervous/non-nervous tissue. As the neo electrode is intended to stimulate the baroreceptors at the media/adventitia border of the carotid sinus (CS) from the outside of the CS, damage to nervous/non-nervous tissue directly adjacent to the electrode would not be considered clinically significant, as long as the integrity of the underlying vessel is maintained, and the therapeutic efficacy when stimulating at maximum tolerable levels before perceptible side effects (pain/local muscle tightening/breathing difficulties due to chemoreceptor activation) is maintained or only modestly reduced.

The testing strategy utilized in this work was heavily informed by the seminal work of Robert Shannon, Douglas McCreery, and Stuart Cogan (McCreery et al., 1990, 1995, 2000, 2010; Shannon, 1992; Woodford et al., 1996; Cogan, 2008), and consisted of multiple benchtop tests to assess electrochemical stability while undergoing long-term stimulation at maximum anticipated levels, along with long-term ovine studies conducted under Good Laboratory Practice (GLP) guidelines. Although benchtop studies are very useful for high resolution characterization of specific electrochemical changes during long-term stimulation, these tests do not as of yet fully recapitulate the complex biological and physical factors that may impact safety in the chronic *in vivo* environment. Consequently, the ultimate test of electrical stimulation for safety is a long-term GLP implant study in an animal model sized to best match the anatomy of the human patient, including post-mortem histology evaluated by an independent and blinded histopathologist. However, it is often infeasible or unethical to stimulate at the maximum parameters one “might need” in a human patient in an animal model due to differences in scale and subject-to-subject variability, as these parameters may cause side-effects in the animal model. In these cases, for chronic animal GLP studies it is commonly accepted to stimulate at parameters just below those that induce side effects, with maximum desired stimulation parameters characterized through supplemental benchtop studies. The high-resolution electrochemical analysis techniques available for benchtop studies also enable more accurate assessment of long-term trends. Moreover, it is common in benchtop studies to use accelerated aging tests (higher frequencies of stimulation, increased temperature, etc.), to mimic years of usage and identify failure modes that may be problematic for a 10-year implant without conducting a 10-year animal study (Takmakov et al., 2015). It is only through detailed electrochemical analysis on the bench combined with animal studies for verification that one can have a reasonable certainty of safety for long-term implantation.

The ultimate goal of both the sponsor and the FDA is patient safety, and there is an understandable desire from both parties to eliminate testing that is costly, time-consuming, and has not been validated for predicting safety in humans. In order to understand the predictive validity in humans, it is critically necessary to make the detailed non-clinical and pre-clinical safety testing that has led to human studies publically available across all neuromodulation products. Further, it is important to assess the validity of these enabling early benchtop and animal safety studies in the context of the eventual safety profile in human patients. These data in turn will lead to the refinement of safety testing strategies to eliminate inefficiencies and improve overall safety. To this end, here we describe the detailed non-clinical and pre-clinical testing conducted to ensure safety of the Barostim neo minimally-invasive electrode for electrical stimulation of the baroreflex prior to human studies. These data were directly used to support necessary approvals from a European notified body and the FDA to conduct human studies in support of market approval.

## Methods

### Benchtop testing

#### Barostim neo neural interface design and justification of parameters for long-term stimulation testing

**Figure 1:**
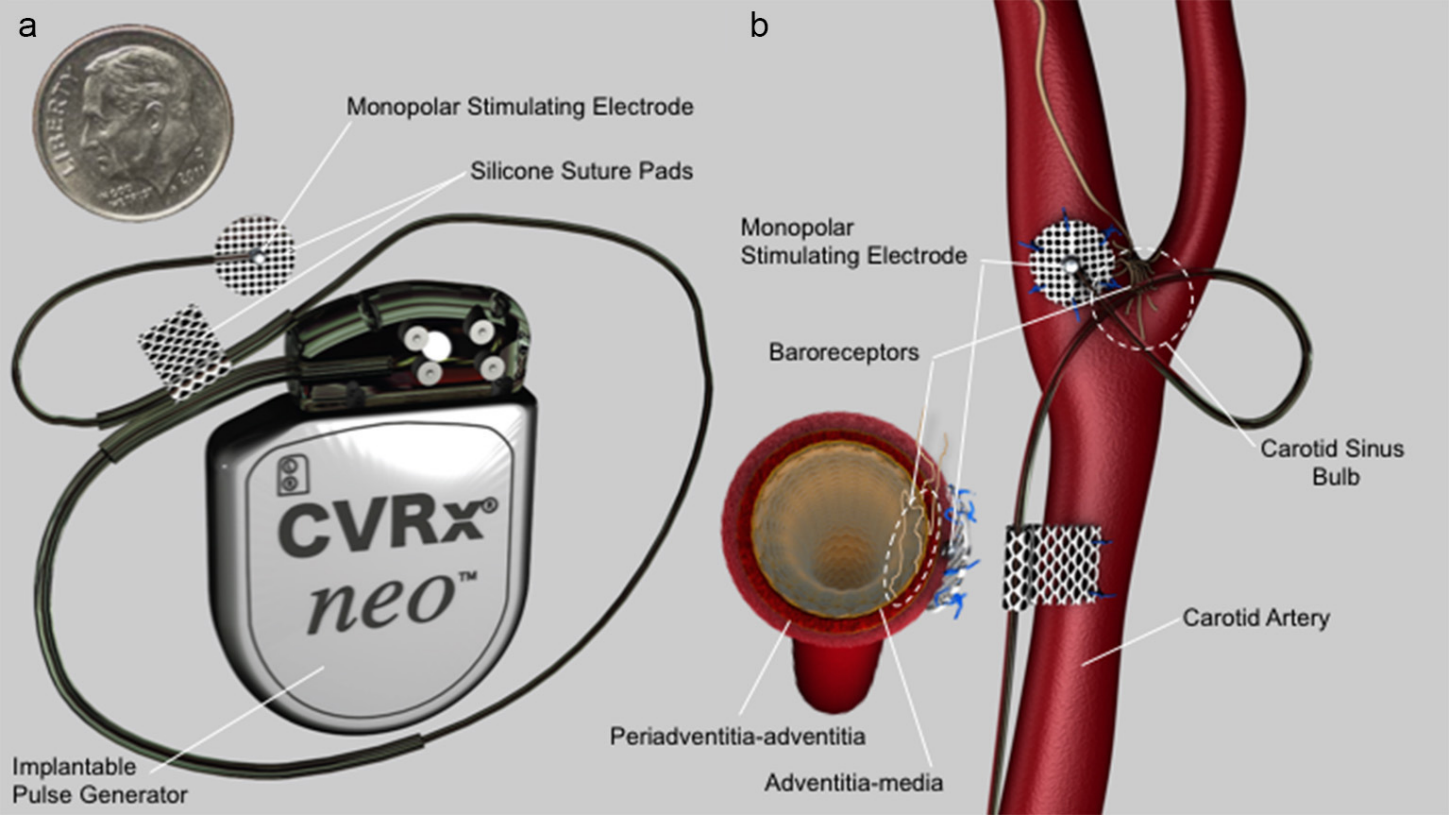
**(a)** Barostim neo electrode assembly and implantable pulse generator (IPG). Electrode consists of a 1 mm diameter platinum/iridium alloy disk coated with a sputtered iridium oxide film (SIROF) which protrudes slightly (100 μm) from a 6 mm diameter insulating backer. **(b)** Electrode configuration and positioning on the internal carotid sinus nerve. Cross-section at the level of the electrode on the lower left side of **(b)** depicts electrode position on adventitia-periadventitia and proud electrode configuration in relation to tissue and baroreceptors.

The Barostim neo electrode used in these studies consisted of a 1 mm diameter Pt/Ir alloy disk (90% platinum, 10% iridium), with a 6 mm diameter insulating backer to help direct current into the carotid sinus bulb as well as aid in suturing onto the bulb for fixation. The electrode was not “flush” with the insulation but “proud,” meaning that the electrode extends ~100 microns from the insulation (see Figure 1). Previous studies have shown that the thresholds needed for activation change dramatically across animal models, as the thickness of the epineurium of the vagus nerve increases when moving from rats to canines to humans, effectively increasing the distance between the epineural cuff electrodes and the fibers the cuff is intended to stimulate (Yoo et al., 2013). Similarly, the baroreceptors are located at the border between the media and adventitia in the artery. As the thickness of the adventitia increases between canines and humans, the distance between the electrode placed on the arterial wall and the baroreceptors was expected to increase.

Initial acute and chronic studies in canine demonstrated consistent baroreflex activation using charge densities up to 60 μC/cm^2^ (60 μs pulse widths, 10 mA, 20–75 Hz). The anticipated increase in thickness of the adventitia when moving from canine to humans was 2–4 fold. As the fall off of an electric field from a monopolar electrode is 1/r where r is the distance from the electrode, we anticipated needing a maximum of ~250 μC/cm^2^. Stimulation parameters tested were at 600 μC/cm^2^ (500 μs pulse widths, 100 mA, 100 Hz) to include a safety factor. For Pt/Ir electrodes, it is a common rule of thumb to limit charge density within a safety threshold of 30 μC/cm^2^ (Cogan et al., 2016). As the estimated charge density at these desired maximum settings was ~600 μC/cm^2^, a sputtered iridium oxide film (SIROF) coating (GreatBatch, Inc., Minneapolis, MN, USA) was applied in the final design to increase the safe limit for stimulation. It is this SIROF-coated electrode that was tested in this work, both on the benchtop and *in vivo*. SIROF and other coatings increase the fractal dimensions of the electrode, and thereby increase the electroactive surface area. By increasing the electroactive surface area, more current can be injected for a given voltage deflection, which may prevent the initiation of irreversible faradaic reactions that may be deleterious to tissue (Cogan, 2008).

#### Long-term stimulation testing

Six neo electrodes were assembled for long-term stimulation testing. Comprehensive initial characterization of the electrodes was performed prior to stimulation utilizing each of the tests listed in subsequent headings to provide a baseline for comparison over the course of long-term stimulation, as well as to identify any issues occurring during shipping/handling/assembly. Long-term stimulation tests were performed in a sealed bottle filled with 250 mL of phosphate-buffered saline (PBS), consisting of 0.73% NaCl and 0.1 M phosphate buffer (pH 7.4) (Thermo Fisher Scientific, Waltham, Massachusetts, USA). 0.2 mg/ml bovine serum albumin (BSA: Thermo Fisher Scientific) was added, and the PBS/BSA solutions were maintained at a temperature of 37°C to more accurately mimic *in vivo* physiological conditions as outlined by Robblee et al. (1980), and commonly used elsewhere (Brummer and Turner, 1977; Rose and Robblee, 1990). PBS/BSA solutions were replaced every third day to minimize degradation of the BSA or formation of bacteria over time.

**Figure 2:**
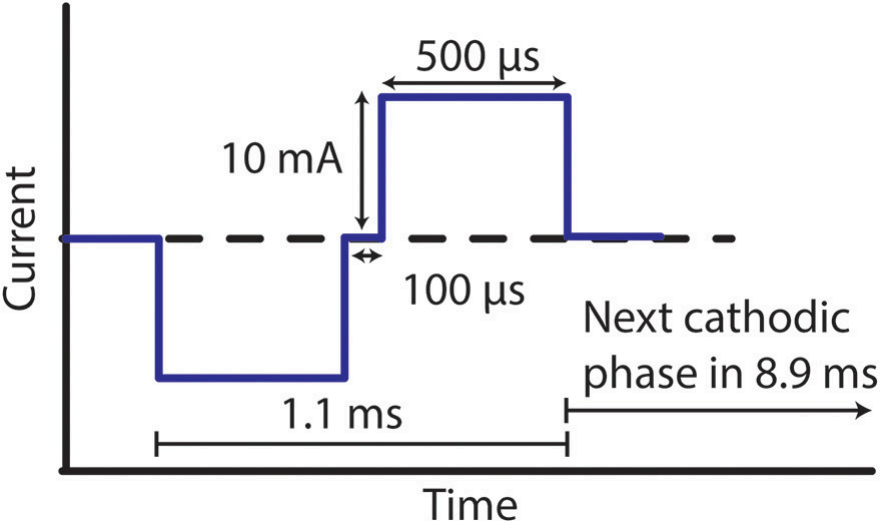
Stimulation waveform used for benchtop testing. Electrodes were stimulated with an amplitude of 10 mA, pulse width of 500 μs, and frequency of 100 Hz.

Over the duration of this study, four of the six electrodes were stimulated, while two electrodes were left unstimulated in PBS/BSA solutions as controls for comparison. A charge-balanced, cathodic-first stimulation waveform of 10 mA, 500 μs, and 100 Hz, was applied to the four stimulated electrodes, resulting in a charge density of ~600 μC/cm^2^. Following the anodic pulse, the cathode and anode were intentionally shorted for 62.5 μs as an additional precaution to minimize electrode polarization. The waveform, depicted in Figure 2, was applied nonstop to the stimulated electrodes, with stimulation paused only for characterization at regular intervals, as described in subsequent sections. For the four stimulated electrodes, the neo electrode was used as the cathode, while a large Pt/Ir coil (exposed surface area >100× the exposed surface area of the neo electrode) was used as the anode. Pt/Ir coils were also placed in solution with the two unstimulated control electrodes. All stimulated and unstimulated electrodes were characterized weekly, as outlined below over the 27-week study, unless specifically noted. In this way, the duration of the benchtop testing was designed to capture phenomena that may occur over the course of the 24-week *in vivo* study.

Each of the benchtop tests listed in Table 1 below was performed at regular intervals during long-term testing, and was intended to provide information about specific, potentially problematic, subtle changes that may have occurred to the Pt/Ir electrode or SIROF coating as a function of long-term stimulation.

**Table 1:** List of benchtop tests performed initially and over long-term stimulation.

**Benchtop Test**	**Rationale**
Visual Inspection at 200× Magnification	Assess changes in visual appearance indicative of dissolution/corrosion as a function of shipping/handling, and long-term stimulation at maximal parameters.
Cyclic Voltammetry	Assess changes in charge-storage capacity of the electrode over time. Identify changes in characteristic oxidation/reduction reactions.
Voltage Transient Measurements	Measure electrode behavior utilizing the clinical stimulation parameters of interest. Assess electrode polarization during therapeutic pulses and compare to theoretical safety limits.
Trace Metal Analysis of Phosphate-Buffered Saline used as Medium for Long-Term Stimulation Testing	Identify trends in dissolution of Pt/Ir over time. Evaluate for levels of dissolution that may be deleterious to tissue or cause early device failure.
Impedance Measurements via Implantable Pulse Generator (IPG)	Provide common frame of reference for measurements available during animal studies.

#### Weekly visual inspection at 200× magnification

##### Experimental set-up

After electrode assembly, but prior to stimulation testing, baseline pictures at 200× magnification under a light microscope were obtained. Initial inspection of these baseline photos enabled a qualitative assessment of coatings for consistency between electrodes, uniformity of coating over the electrode surface, handling damage, and insulation overlap. During long-term stimulation testing, 200× pictures were taken weekly to enable a qualitative assessment of electrode coating delamination/dissolution and obvious discoloration indicative of a change in the material surface. Efforts were made by the microscope operator to orient each electrode similarly from week to week to facilitate comparisons across weeks.

##### Rationale

Light microscopes are often readily available, inexpensive, and easy to use for weekly data collection. In comparison to electrochemical testing detailed in subsequent sections, which represent an average of electrochemical behavior across the electrode surface, light microscopy allows the operator to evaluate location-specific gross changes in coating appearance that may not be detected by more sensitive electrochemical assays. Given these data are supplemental to more sensitive electrochemical tests—and based off of prior successful regulatory submissions for electroactive neural interfaces—it was not deemed necessary to obtain high-resolution images of the electrode surface using scanning electron microscopy (SEM), which can also suffer from the same operator variability and subjectivity in interpretation as light microscopy. As the costs for external third-party SEM analysis decreases over time, SEM may provide more detailed information about subtle changes to the SIROF coatings and the underlying Pt/Ir substrate at more reasonable cost.

##### Success criteria

The primary metric of success is a subjective evaluation of electrode appearance at 200× magnification after 6 months of stimulation, in comparison to pre-stimulation baseline and identically treated “control” electrodes not subjected to stimulation. Given 6 months of stimulation at levels in excess of those anticipated for clinical use, “failure” would be indicated by visually evident changes in appearance of the coating at the electrode/insulation border that would suggest a progressively increasing material failure. Moreover, this progressive failure would need to be to an extent that one would anticipate clinically significant tissue damage over the remainder of the 10 year lifetime or a failure to maintain reasonable therapeutic efficacy during the same period.

#### Weekly electrode cyclic voltammetry testing

##### Experimental set-up

Cyclic voltammetry (CV) was performed as a three-electrode measurement in which the potential of the neo electrode with respect to a noncurrent-carrying saturated calomel electrode (SCE) reference was swept cyclically at a constant rate of 50 mV/s between −0.547 and 0.847 V while allowing current to flow between the neo electrode and a large Pt/Ir coil counter electrode (sweep ranges were slightly adjusted to account for the open circuit potential of SCE). The potential provided the driving force for reactions at the electrode interface, while the faradaic current was proportional to the rate of these reactions. CV identifies the presence of stereotypical electrochemical reactions occurring at the electrode (primarily an Ir^3+^ ↔ Ir^4+^ reaction for a SIROF coated electrode) and provides information on the reversibility of the reactions, the quantity of electroactive material on the electrode, and the stability of the electrode (Cogan, 2008). CV measurements were performed in 0.1 M PBS buffer, as well as 0.9% NaCl solution for comparison with CVs taken at GreatBatch, Inc., immediately after application of the SIROF coating.

##### Rationale

It has become common practice to characterize stimulation electrodes by their cathodal charge storage capacity (CSC_c_). The CSC_c_ is calculated from the time integral of the cathodic current in a slow-sweep-rate CV over a potential range that is just within the water electrolysis window. Electrolysis of water at the electrode interface has been proposed as a primary driver of stimulation-induced tissue damage. As the potential immediately prior to water electrolysis is often set as the safe maximum limit for stimulation, the CSC_c_ is essentially a measure of the total amount of charge safely available for a stimulation pulse (Cogan, 2008; described further in Supplementary Methods). Moreover, the size and shape of the CV provide detailed information about the electrochemical behavior of the neural interface over time. Problematic changes in the nature of the neural interface due to dissolution/corrosion of the SIROF or underlying substrate over time would be expected to result in obvious changes in the area under the CV curve and the locations/shapes of faradaic peaks generated by oxidation or reduction reactions at the electrode surface.

##### Success criteria

For an electrode to pass this testing, its CSC_c_ should become stable after several weeks of stimulation, indicating no progressively increasing changes to electrochemical behavior. The electrode CSC_c_ magnitudes should indicate that charge density for maximum stimulation levels remain within safe maximum limits. There should be no changes in the size and shape of the characteristic SIROF CV that would suggest a change in the nature of the neural interface as subjectively assessed by an expert.

#### Periodic electrode voltage transient measurements

##### Experimental set-up

The voltage transient measurement set-up was identical to that described above for long-term stimulation testing, with the exception that an oscilloscope was used to measure the voltage transient generated across the cathode/electrolyte/anode system during stimulation. Here, the Pt/Ir coil anode was 200× the exposed surface area of the neo cathode so that charging of the return electrode was minimal, and therefore the Pt/Ir coil was used as both the return electrode and reference electrode.

##### Rationale

Although CV provides a sensitive measure of the electrochemical behavior of an electrode surface, electrochemical behavior is known to change as a function of current amplitude and waveform applied (Cogan, 2008). Voltage transients are used to determine if polarization on the electrode itself during maximal stimulation pulse amplitudes/widths/frequencies exceed the level necessary to cause the electrolysis of water. This potential extreme was compared with established potential limits, beyond which are considered unsafe due to polarization of the electrode. For SIROF coated electrodes, the established maximum cathodic potential, E_mc_, is −0.6 V (Cogan, 2008). As long as E_mc_ is between 0 and −0.6 V, stimulation at the applied therapy parameters will not cause water electrolysis, and therefore is not anticipated to result in significant tissue damage or electrode dissolution during a lifetime (10 years) of stimulation (Cogan, 2008; Described further in Supplementary Methods).

##### Success criteria

E_mc_ should remain under −0.6 V over the 6 months of long-term stimulation testing. No progressive trend should be evident that may suggest E_mc_ would increase to levels beyond −0.6 V if stimulation testing were continued for 10 years.

#### Periodic trace metal analysis of PBS/BSA solutions for iridium and platinum dissolution by inductively coupled plasma mass spectroscopy

##### Experimental set-up

PBS/BSA solutions that were used for stimulation and control electrode sets were acquired and stored for assay analysis. Samples were obtained every 2 days, stored weekly through 9 weeks (see Methods: Long-term stimulation testing), and monthly after week 9. The samples were sent to Legend, Inc. to perform inductively coupled plasma mass spectroscopy (ICP-MS). ICP-MS allowed quantification of platinum and iridium/iridium oxide electrode material that aggregated in solution with a detection limit of 12.5 ng of material.

##### Rationale

The primary cause of stimulation induced tissue damage is still a topic of debate. The two primary theories are cell death brought on by cells being unnaturally stimulated for long periods of time (hyperactivity, excitoxocity) and toxic products due to electron transfer processes (electrochemical reactions Kumsa et al., 2016). Although the exact levels of platinum or iridium particulates resulting from chronically implanted electrode dissolution processes necessary to cause cell death are unknown, a common frame of reference is an early study by Rosenberg, et al. characterizing the inhibition of cell division in escherichia coli by electrolysis products from a platinum electrode (Rosenberg et al., 1965). These data suggest that physiologically relevant concentrations of platinum dissolution products—although not necessarily problematically damaging—are in the range of 1–10 parts per million (ppm).

Prior studies have demonstrated that the inclusion of physiologically relevant amounts of human serum albumin (HSA) reduces rates of dissolution by limiting the movement of products/reactants from the electrode surface (Robblee et al., 1980). Similarly, encapsulating tissue endemic of chronic implants further restricts Pt primarily to the encapsulating tissue (Robblee et al., 1983). As these assays were performed at maximum therapy settings in PBS/BSA, and the relevant electrode literature indicates that rates of dissolution should decrease over time (Brummer and Turner, 1977; Robblee et al., 1980, 1983), these estimates should be considered on the high side of what would be expected *in-vivo*.

##### Success criteria

The overall amount of Pt/Ir dissolution caused by stimulation should remain under the 1–10 PPM range projected for a 10-year lifetime. Dissolution measurements should not suggest the amount of material lost would create a significant deterioration of electrode performance. No progressive trend in Pt/Ir dissolution over time should be evident that would indicate that study results would not extrapolate to a 10-year implant period.

#### Weekly electrode implantable pulse generator (IPG) impedance measurements

##### Experimental set-up

Electrode impedance can change dramatically as a function of applied current and electrolytic medium (Cogan, 2008; Wei and Grill, 2009). To more accurately mimic the *in vivo* electrode-tissue interface during application of BAT, impedance was calculated by measuring the voltage during charge balanced constant-current stimulation (10 mA, 500 μs, 100 Hz) in PBS/BSA solution. Specifically, the CVRx IPG calculated impedance by measuring the voltage at 31.25 μs before the end of a pulse and dividing it by the applied current. Due to the fact that impedance is calculated by measuring the voltage at a specific instant in time resulting from a single constant current pulse, slight variability between measurements is expected. Any voltage present at the electrode—even if it is not related to the tissue impedance, but is instead related to electrode polarization—will have a direct impact on the reported impedance. These measurements were only taken for stimulated electrodes to limit the charge applied to unstimulated controls, and were obtained every 2 days of stimulation. At the 20 week mark the initial IPGs ran out of battery at maximal stimulation parameters and were replaced with new IPGs. As the impedance measurements taken by an IPG also include the internal impedance of the IPG itself, and the internal IPG impedance can vary between IPGs, impedance data was normalized to the mean impedance of a particular electrode/IPG combination.

##### Rationale

*In vivo* impedance measurements can only be taken with the IPG. As such, benchtop impedance measurements using the IPG during long-term stimulation are the most useful frame of reference in comparison to impedance measurements taken *in vivo* over time to isolate electrode specific changes. Additional electrochemical impedance spectroscopy measurements were also obtained, which should be viewed as redundant confirmation to IPG impedance measurements, but obtained using applied sinusoid waveforms at much lower voltages (see Supplementary Materials).

##### Success criteria

IPG impedance measurements over time should remain stable during long-term stimulation.

### *In vivo* chronic study

#### Subjects

The ovine model was selected for chronic animal studies to evaluate safety of the full neo implantable system, as the ovine carotid arteries are similar in diameter to the human carotid artery, which enables accurate assessment of wear and tear due to geometry. Surgical procedures and data analysis for the ovine studies were conducted by American Preclinical Services, LLC and adhered to GLP guidelines. The Institutional Animal Care and Use Committee (IACUC) review board at American Preclinical Services, LLC approved all of the outlined procedures for this study. The subject group consisted of seven male crossbred sheep between 100 and 150 kg. Two cohorts were implanted with the full neo system, with three animals belonging to a 12-week survival group and four animals belonging to a 24-week survival group. Data from the 12 and 24-week cohort were used to support the CE mark and FDA IDE to conduct the Pivotal Trial (equivalent to a Phase III drug study). A 24-week end-point was requested by the FDA and is a common end-point across the neuromodulation industry, as it represents a point in time after the implant has reached steady-state *in vivo* and allows sufficient time to identify electrode degradation issues.

The neo IPG has the capacity to stimulate two different neo electrodes simultaneously. Each animal was implanted with a single IPG and four neo electrodes and associated leads, with two electrode-lead assemblies connected to the IPG and two unconnected to serve as unstimulated controls. The 6 mm “backer” for the electrode used for insulation to direct current preferentially into the adventitia was constructed out of suture pad material. Four to six 6–0 prolene sutures were placed evenly spaced around the electrode backer to secure the electrode to the vessel. Prior to enrollment, each animal underwent transcutaneous ultrasound screening of their proposed implant sites to confirm both left and right common carotid artery diameters were ≥7.0 mm. Once enrolled, the left and right common carotid arteries were exposed and two neo electrodes were implanted on each artery; one electrode from each side was randomly selected and connected to the IPG. Subsequently, post-implant carotid angiograms were taken to confirm that there were no adverse results of the implant on carotid blood flow. At the conclusion of the implant procedure, the animals were recovered in their kennels and maintained for their predetermined survival lengths.

#### Study design

Each electrode that was connected to the IPG at implant was stimulated continuously throughout each animal's in-life survival period. IPG stimulation settings were set at the maximum current amplitude that could be applied at a frequency of 20 Hz and pulse width of 250 μs without any observable side-effects to the animal. The most common side-effect limiting use of higher stimulation parameters was slight neck-strap muscle fasciculation, which was only detected through careful direct external palpation of the animal's neck. Maximum currents before side effect varied from 0.735 to 8 mA, which compares favorably to data from existing vagal nerve stimulation tripolar cuff electrodes (FDA, 1998). While occasional downward adjustments in pulse amplitude were needed to avoid neck-strap muscle fasciculation, tolerable stimulation limits were very consistent over the timeframe of the experiment. Once a tolerable threshold was determined, the ability to increase settings over time was not explored. Across both cohorts, 28 electrodes in total were implanted with 14 electrodes continuously stimulated through the duration of the animal study. Overall animal health following implantation was recorded daily, along with impedance measured to ensure electrical conductivity of the neural interfaces was maintained (Described further in Supplementary Methods).

#### Histological analysis

At the conclusion of each animal's survival period carotid artery angiograms were taken followed by euthanasia and transference to necropsy for gross assessment and target tissue procurement. Electrode integrity was inspected after explantation, and implant sites were microscopically evaluated by an independent histopathologist. The evaluation was focused on describing the gross and cellular response of the carotid arteries to the chronic implantation of the neo electrode with and without electrical stimulation. Each animal's head and neck complex was fixed as a single unit via the arterial system. First, the complex was perfusion flushed with Lactated Ringers' solution for a period of 18 to 36 min. Next, the complex was perfusion fixed with 10% neutral buffered formalin (NBF) for a period of 17 to 25 min. Once fixed, artery, nerve, and electrode complexes were dissected free via scalpel dissection and electrocautery, followed by immersion in 10% NBF. The tissue samples were dehydrated in a graded series of alcohols, embedded in paraffin, and microtomed to thin sections for slide preparations.

For histological analysis, Movat's staining was conducted on axial tissue slices. If neointimal hyperplasia was present, the severity of the hyperplasia was assessed. Minimal was defined as 2–5 cell layers in thickness and mild was defined as 6–10 cell layers in thickness. Either minimal or mild neointimal hyperplasia would not be considered of clinical significance (not resulting in clinically significant stenosis). Inflammation was designated as granulomatous if the infiltrate included multinucleated giant cells as well as macrophages and lymphocytes. Inflammation was designated as mononuclear cell if macrophages, lymphocytes and/or plasma cells were present. Gross assessment was also performed on all major organs (Described further in Supplementary Methods). Movat pentachrome stain was used to histologically evaluate elastic fibers, cell nuclei, collagen, reticular fibers, mucin, and fibrin.

Success criteria for this study included a summarized report from an independent histopathogist indicating that: (1) any change in inflammation/tissue health in the vicinity of the electrode due to stimulation was clinically insignificant, (2) integrity of the underlying carotid vessel was maintained, and (3) end-organ assessments at term were normal. Further, an independent report was required from the contract research organization indicating that no clinically significant stimulation induced adverse effects were observed throughout the duration of the animal study.

## Results

### Visual electrode analysis over long-term stimulation

**Figure 3:**
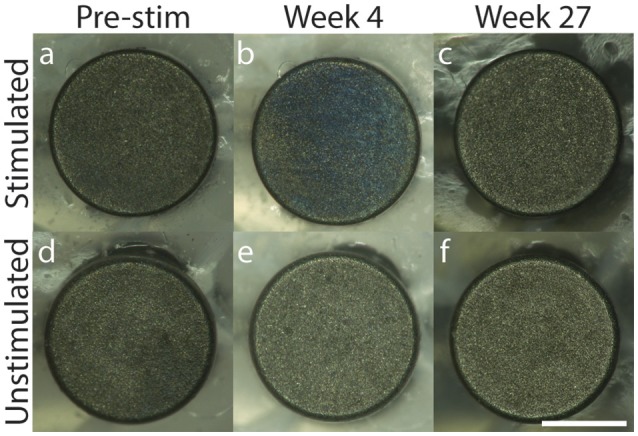
200× microscopy of representative stimulated **(a–c)** and unstimulated **(d–f)** electrodes across 27 weeks with pre-stimulation images in **(a,d)**, Week 4 images in **(b,e)** and Week 27 images in **(c,f)**. Scale bar = 0.5 mm.

For all electrodes tested, 200× microscopy pictures taken each week over the duration of stimulation demonstrated no delamination of the SIROF coating over time, and no discoloration of the underlying electrode substrate that would be indicative of unwanted electrochemical reactions, such as hydrolysis (Figure 3; weekly images for representative stimulated and unstimulated electrodes can be found in Supplementary Figures 4 and 5, respectively). Minor blue discoloration was observed on the surface of the stimulated electrodes only (Figure 3b), but gentle rinsing with deionized water removed the discoloration without damaging the SIROF coating. Both stimulated and unstimulated electrode groups were rinsed with deionized water every 4 weeks, beginning on week 6. It is likely that BSA protein aggregation on the surface of the stimulated electrodes caused the blue discoloration as the BSA protein added to the solution was blue in color.

### Implantable pulse generator impedance measurements

**Figure 4:**
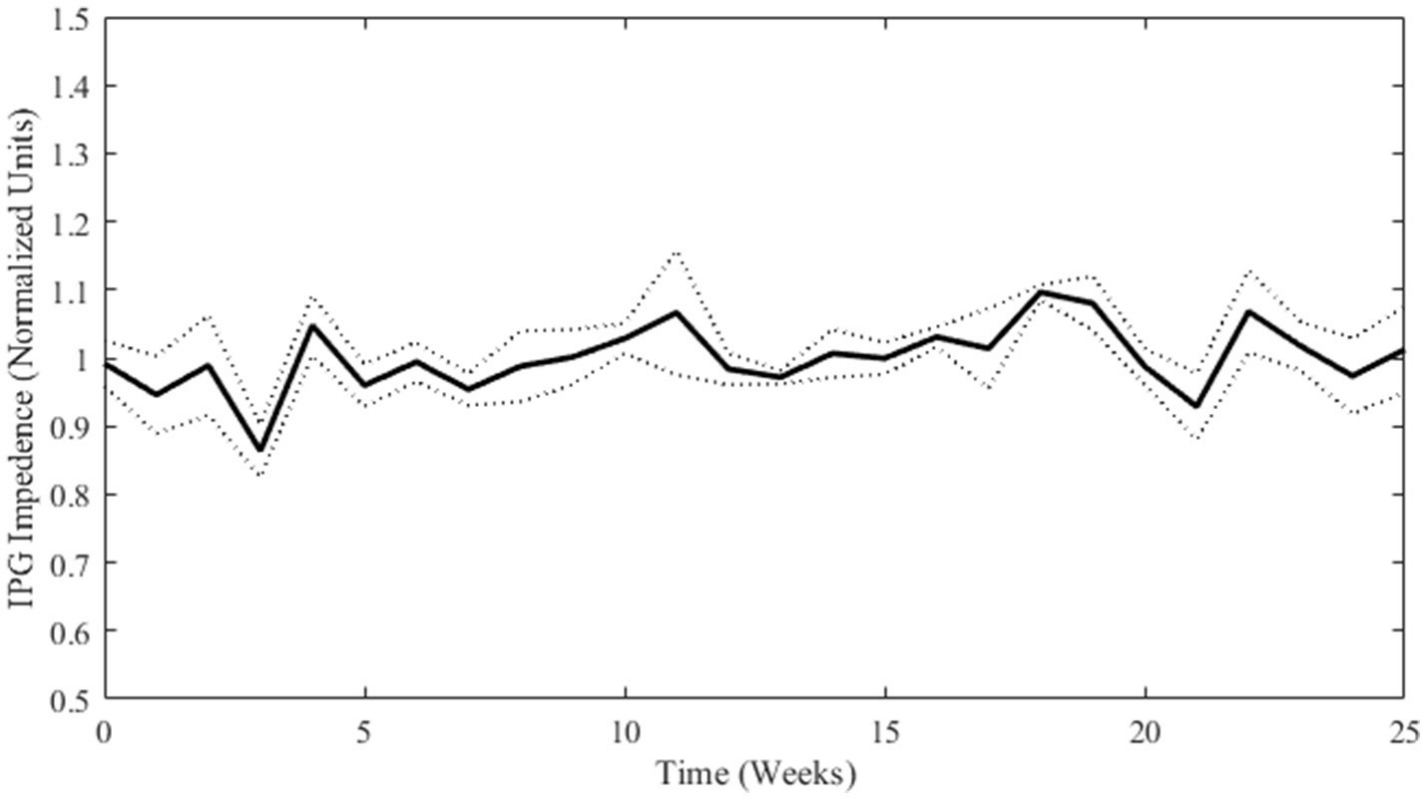
Normalized impedance (mean ± *SD*; *n* = 4) over time for stimulated electrodes as measured by the IPG. Weeks 0 through 12 are not statistically different from Weeks 13 through 25 (student's *t*-test *p-*value = 0.15).

If significant dissolution of material or delamination of the SIROF coating occurred, an obvious decrease (dissolution) or increase (delamination) in impedance at therapeutic stimulation pulse parameters would be expected. As the IPG battery depletes over time, slight variability in impedance measurements are to be expected, but more drastic changes would be indicative of electrode damage. As can be seen in Figure 4, no deleterious changes in electrode material or performance were evident from the weekly impedance measurements (see Supplementary Figure 6 for individual electrode measurements). The IPGs had to be replaced on week 21 due to a depleted battery. To account for variation in the intrinsic impedances of the IPGs themselves, all values are normalized to the mean impedance of a particular electrode/IPG combination. The normalized values for individual electrodes were averaged for each week and the standard deviation was calculated. Data from weeks 26 to 27 were not properly collected and so no data is shown for those time points.

### Deactivation of iridium oxide coatings in air

Sputter coating iridium oxide onto the surface of an electrode creates a hydrated oxide film that greatly increases the electrode's ability to inject charge by a fast and reversible faradaic reaction involving reduction and oxidation between Ir^3+^ and Ir^4+^ states of the oxide (Cogan, 2008). When left in air or left unstimulated in solution, the hydrous oxide film undergoes a process known as “deactivation” (Loeb et al., 1995; Robblee et al., 1995). Deactivated electrodes revert to CVs and impedance spectrograms that are typical of clean but inactivated iridium (Loeb et al., 1995). Deactivated SIROF coatings can be “reactivated” simply by immersing the electrode in an electrolytic medium and applying either multiple CVs, or charge balanced pulses, to drive repeated oxidation and reduction of the Ir. As neo electrodes were exposed to air and left unstimulated during shipping and assembly, initial measurements were taken in a more deactivated state. After applying charge balanced therapy for 48 h, the SIROF became mostly activated, and reached a stable point of full activation between 1 and 3 weeks of stimulation. The CSC_c_ for all deactivated electrodes was >2 mC/cm^2^. The conservative estimate of 10% of the CSC_c_ suggested by Cogan et al. to predict safe charge density limits for neural stimulation, yields a limit of 200 μC/cm^2^ per pulse (Cogan et al., 2016). The maximum therapy settings (10 mA pulse amplitude, 500 μs pulse width, 100 Hz) correspond to a charge density of ~600 μC/cm^2^ per pulse, which exceeds the estimated safety limit for electrodes in a deactivated state. However, the waveform that exceeds the limit also causes the electrodes to become activated, thereby raising the safety limit. The CV measurements show that the safety limit was much higher in week 1, and the assay results show that there was not unusual delamination or dissolution during the period of time during which the electrodes were becoming activated.

### Cyclic voltammetry measurements

**Figure 5:**
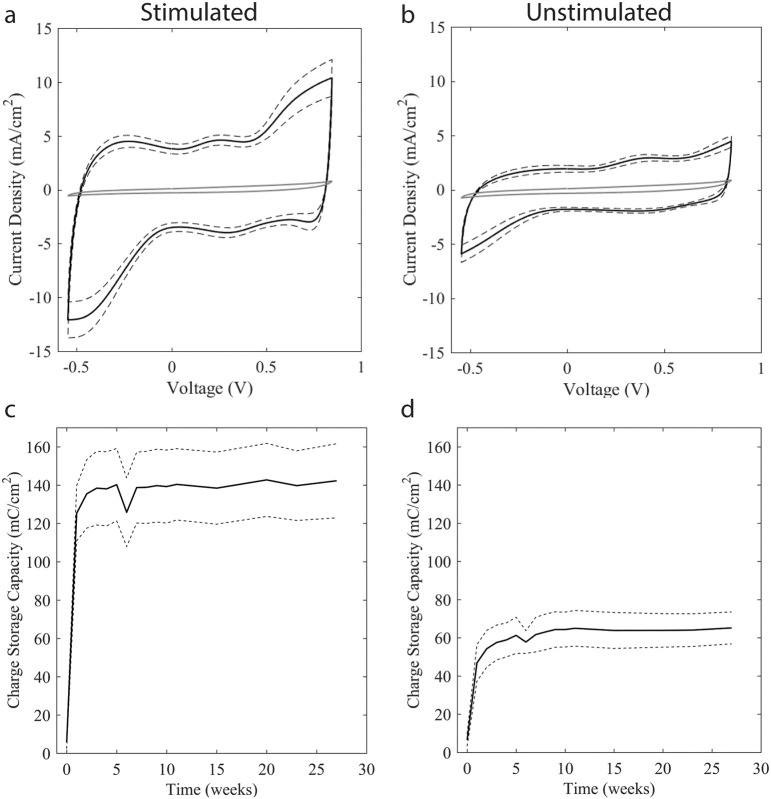
CV and accompanying CSC_c_ measurements across testing weeks. CVs taken upon receipt are shown in solid gray in **(a,b)**, with black lines representing the mean (solid black) and standard deviation (dashed black) of CVs collected between weeks 1–27 of stimulation. CSC_c_ was calculated from CV measurements with mean (solid black) and standard deviation (dashed black) shown for stimulated **(c)** and unstimulated **(d)** electrodes. Due to the unstimulated interval between SIROF application at Greatbatch and assembly at CVRx, SIROF coatings became deactivated, resulting in a dramatically reduced CSC_c_ as seen in the as-received CV taken prior to stimulation in both **(a,b)**. After 48 h of charge-balanced stimulation at maximum therapy settings, the SIROF coating regained most of its original activation. By week 3, CVs were nearly identical in form to the example CVs taken at Greatbatch. Weeks 8 through 11 are not statistically different from Weeks 15 through 27 for either stimulated or unstimulated electrodes, student's *t*-test *p*-value 0.300 and 0.925, respectively, indicating stability. Location and magnitude of the peaks on the CV indicate Ir^3+^ ↔ Ir^4+^ oxidation and reduction reactions stereotypical for SIROF coatings.

CVs of the neo electrodes taken at Greatbatch immediately after application of the SIROF coating demonstrate characteristic current peaks indicative of the Ir^3+^ ↔ Ir^4+^ reversible reaction and large CSC_c_ values exceeding 100 mC/cm^2^ (Supplementary Figure 7). Figure 5 depicts the CVs taken in 0.1 M PBS buffer after shipping and assembly of the neo electrodes, prior to stimulation, and after each week of testing. Prior to stimulation, the CSC_c_ of all electrodes with inactivated SIROF was <10 mC/cm^2^ (Supplementary Table 1), but still sufficient to ensure safe stimulation at therapeutic settings. The stimulated electrodes became fully activated after 1–3 weeks of stimulation, as indicated by the dramatic increase in CSC_c_, (Figure 5c). After 2–3 weeks of stimulation the CV measurements became very stable and consistent, demonstrating that electrochemical changes at the electrode surface after the SIROF coating became fully activated were insignificant. The unstimulated electrodes recovered a smaller degree of SIROF activation simply through the cycling required to obtain the CV measurements.

### Voltage transient measurements

**Figure 6:**
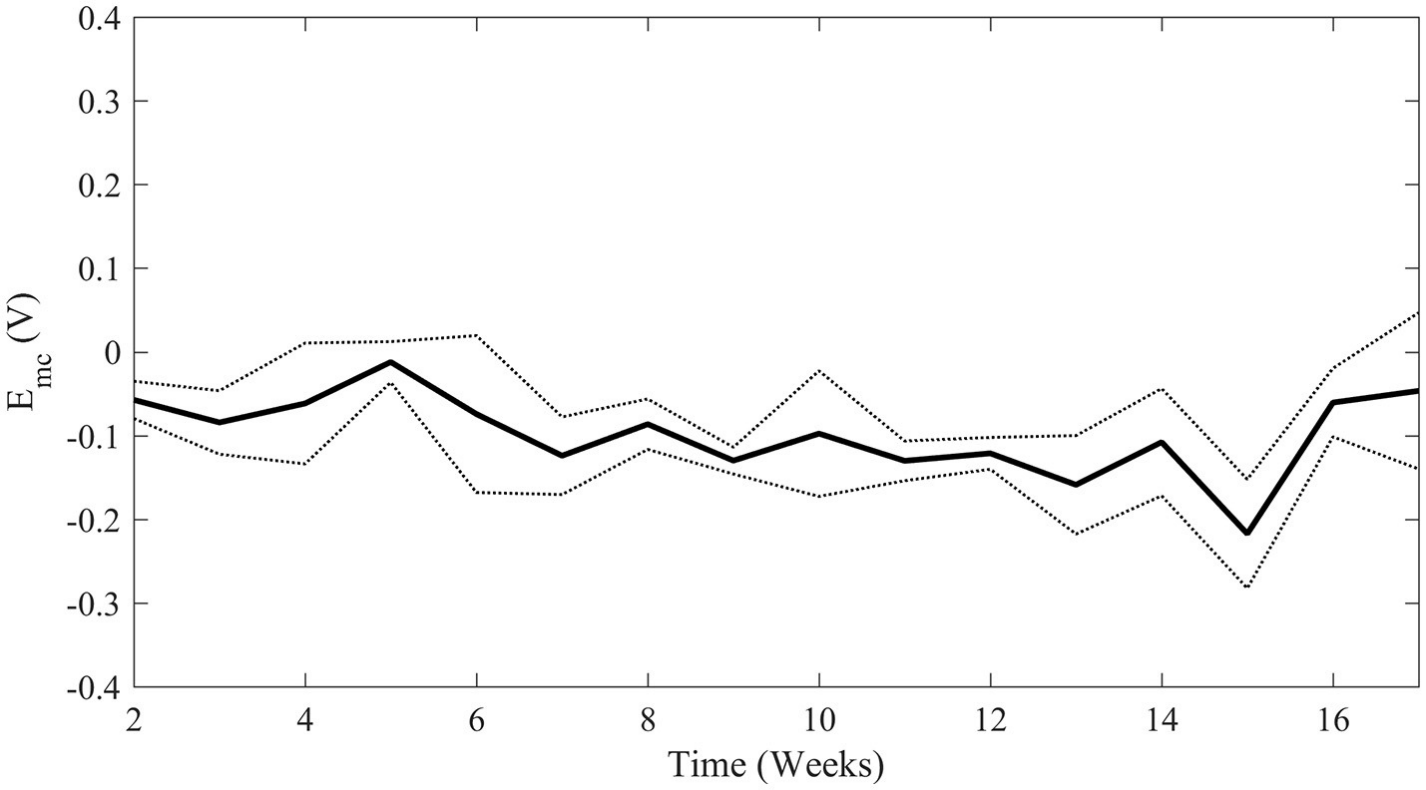
Average maximum cathodic polarization, E_mc_, over time of all four stimulated electrodes. The solid line represents mean E_mc_ with standard deviation represented by the dotted lines. Weeks 2 through 8 are not significantly different than Weeks 9 through 17 (student's *t*-test *p-*value = 0.133) indicating stability above the safety limit of −0.6 V.

Maximum cathodic potential was calculated from weekly voltage transient measurements. Since the Pt/Ir coil acted as both the reference and return electrode, the equilibrium potential E_e_ represents the difference in half-cell potentials between the Pt/Ir coil and the SIROF-coated neo electrode. Additionally, as testing was conducted in a solution containing BSA, it would be expected that the open circuit potential of the Pt/Ir coil would be more negative than usual, as has been shown to be the case with other metal electrodes (Karimi et al., 2011; Hedberg et al., 2013). The average calculated E_mc_ over 17 weeks of stimulation is shown in Figure 6. In all cases, E_mc_ was well within the established safety limit of −0.6 V.

### Loss of iridium and platinum into solution over time during long-term stimulation

**Figure 7:**
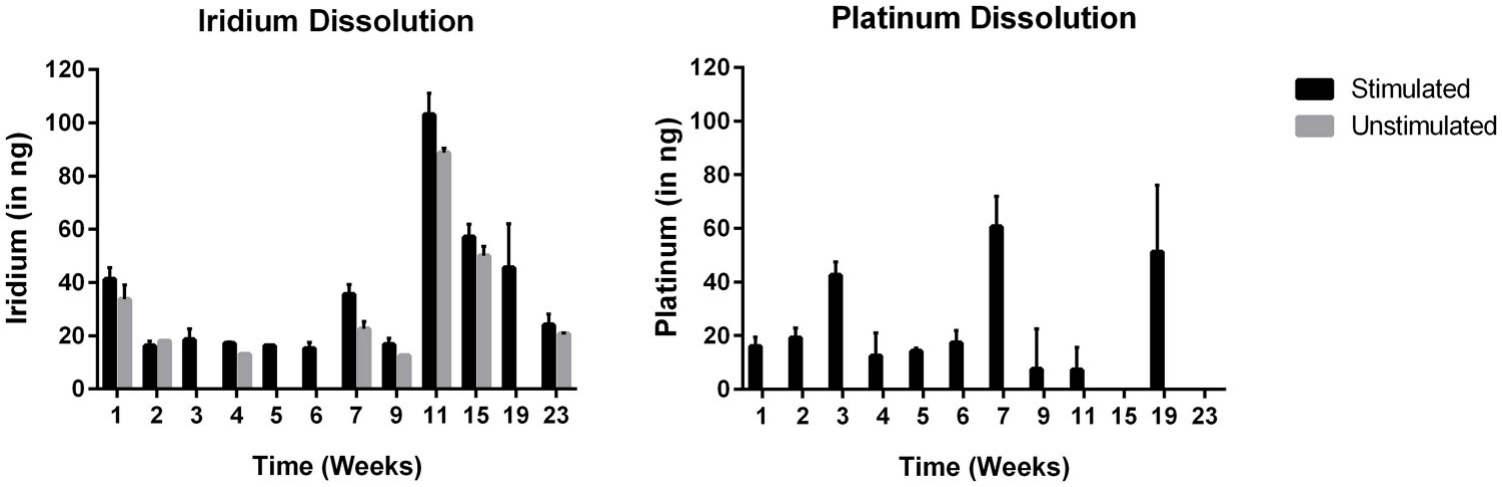
Loss of iridium and platinum into solution over time during long-term stimulation (mean ± *SD*; *n* = 4 stimulated; *n* = 2 unstimulated). Note that every 4 weeks starting on week 6, a build-up of what is presumed to be BSA that had aggregated on the electrode surface was removed by spraying the surface of each electrode lightly with deionized water.

Figure 7 depicts the loss of iridium and platinum into solution over time per electrode. In unstimulated electrodes, the amount of platinum lost to solution was below detection limits, as expected. Small amounts of weakly adhered SIROF is expected after the sputter coating process, and the higher levels of iridium in solution during week 1 is a likely result of this weakly adhered SIROF diffusing into solution. The small amounts of Ir lost over time as determined by ICP-MS measurements are barely above the detection limits for ICP-MS testing (12.5 ng) and can be considered trivial. The putative protein coating that had built up on the electrode surfaces over time was removed by lightly brushing the surface with deionized water on a monthly basis beginning at week 6. As predicted by Robblee et al. (1983), removing the putative protein coating may have been partially responsible for the transient increase of Pt and Ir dissolution in week 7, and weeks thereafter when routine cleaning with deionized water was implemented as part of the protocol. An alternative explanation is that the light brushing of the surface with deionized water creates a mechanical stress that can lead to future small increases in loss rate. The fact that the unstimulated electrodes began to show an increase in Ir lost into solution beginning at week 7 support this possibility as well.

The maximum amount of combined platinum and iridium lost into solution over a 2-day interval by an electrode stimulated at maximum therapy settings was <110 ng. These results are consistent with the data previously presented and with calculated safety limits using established techniques for voltage transient analysis. Even taking this maximum material loss for a 2 day interval and extrapolating forward, the total amount of material that will be lost over a 10-year lifetime is ~0.2 mg, which is <2% of an electrode's original mass of 11 mg. Over the lifetime of an implant, 0.2 mg of material lost is well beneath the levels that would result in deterioration of electrode performance, equating to 0.2 parts per million if all of the material were restricted to tissue within 1 cm from the electrode surface. It is worth noting that extrapolation of data from cell culture to an *in vivo* setting is imperfect given the inherent environmental differences. Even if all of the dissolved material were restricted to tissue directly in contact with the electrode and therefore reaching physiologically-relevant concentrations (1–10 ppm), the majority of material would be physically confined by the fibrous capsule encasing the implant and access to healthy tissue would be restricted. As Pt/Ir dissolution measurements were taken at maximum therapy settings and then the single worst measurements extrapolated to 10 years, material dissolution is a self-limiting process over time (Robblee et al., 1983; Donaldson and Donaldson, 1986), and fibrous encapsulation of the implanted electrode is more extensive than films formed with BSA, lifetime estimates of material lost obtained via benchtop testing should be considered very conservative worst-case scenarios.

### *In vivo* chronic study

#### Microscopic histological analysis

**Figure 8:**
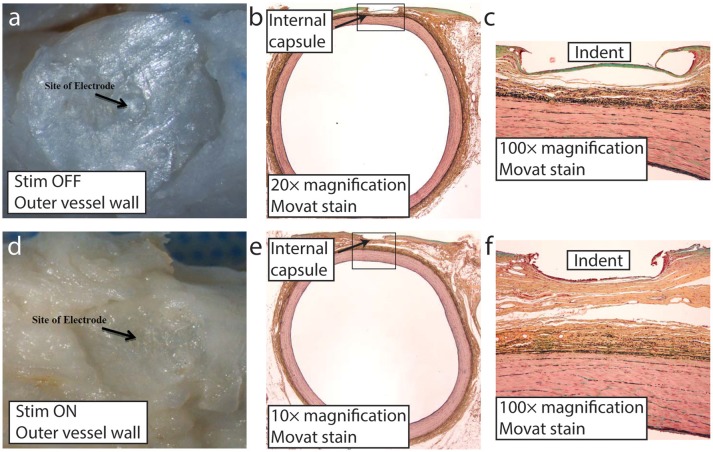
Representative images of the neo implant site (stimulation on and off) at 24 weeks post-implant. Distinct impression from the proud electrode is visible on the outer vessel wall following implant removal at the time of microdissection with stimulation off **(a)** and on **(d)**. Low magnification of the carotid artery Movat stained section showing the thin internal capsule that separated the implant from the underlying vessel wall with stimulation off **(b)** and on **(e)**. Higher magnification (100×) with stimulation off **(c)** and on **(f)**. The indent labeled in **(c)** and **(f)** is the result of the proud electrode, which by design protrudes ~100 μm from the insulating backer.

At the time of necropsy, all of the electrodes were found to be sutured securely in place, although some of the electrodes were slightly better apposed than others to their respective carotid implant sites. According to the independent, third party histopathologist, there was no evidence of infection or clinically significant tissue responses associated with any of the implant sites. The implants were fully encapsulated and all of the common carotid arteries appeared normal (Supplementary Figure 11). Movat pentachrome stain was used to histologically evaluate elastic fibers, cell nuclei, collagen, reticular fibers, mucin, and fibrin. Microscopically, there was no evidence of erosion, thrombosis, or stenosis at any of the implant sites at 24 weeks. Furthermore, the arterial implant sites all appeared completely endothelialized and oval to round in shape. All of the electrode capsules were normal. Areas of granulomatous inflammation were seen primarily at the interface of the silicone backer and the surface of the internal capsule (Figure 8). On occasion, minimal granulomatous inflammation was seen subjacent to the electrodes, typical of a foreign body reaction elicited by chronic implantation of a device within the body. Lastly, there were no gross or microscopic differences seen when comparing the findings from the electrically stimulated implant sites to those of the unstimulated implant sites. As seen in Figure 8, a slight depression in the thin internal capsule was typically seen directly subjacent to the neo electrode, following the configuration of the electrode which extends 100 microns from the insulating backer. Minimal neointimal thickening was discernible in the Movat's-stained low magnification images. Finally, the independent report from the contract research organization indicated that no clinically significant stimulation-induced adverse effects were observed throughout the duration of the animal studies.

#### Visual and electrical post-dissection electrode evaluation

Post-dissection, electrodes were visually inspected via light microscopy, and electrically inspected with a digital multimeter. Visually, there was no evidence of damage (dissolution or degradation) on any of the electrodes and the electrode backers appeared intact. Based on DC resistance measurements, all electrode-lead assemblies were shown to have electrical continuity. Three electrodes from the 12-week group, and four electrodes from the 24-week group were noted at the time of dissection as not being fully apposed to their respective carotid implant sites. In all cases, this is presumed due to an overly conservative surgical implantation technique—not tying the sutures to the periadventitial wall tightly enough—as opposed to the result of an adverse biological reaction from the presence of the electrode, or from electrical stimulation (Supplementary Figure 12). Throughout the 12 and 24 week in-life durations, none of the electrodes experienced performance failures, and none elicited a negative health or safety response within their respective test systems. Furthermore, there was no evidence of infection at any of the 12 or 24 week electrode implant sites. Lastly, none of the explanted electrodes appeared physically or electrically damaged upon visual and electrical inspection. When taking into consideration the results of this study at the conclusion of a 12 and 24 week implant period, the electrode met the success criteria of the protocol specified endpoints: overall animal health, tissue response to the electrode with and without stimulation indicating no clinically significant tissue damage, and electrode performance.

## Discussion

### Aggregation of bovine serum albumin on stimulated electrodes—implications

Preferential aggregation of what is presumed to be BSA was noted on all stimulated electrodes during long-term stimulation testing, whereas BSA aggregation was not visibly evident on unstimulated controls. Preferential aggregation of protein on stimulated electrodes in comparison to unstimulated controls has previously been noted in an *in vivo* study conducted by Weiland et al (Weiland and Anderson, 2000). One possible driver for the preferential aggregation of charged proteins on the stimulated electrode is the observed voltage offset that developed between the neo electrode (cathode) and the large Pt/Ir coil (anode) during constant current stimulation. These offsets have been observed in other studies of deep brain stimulation and cochlear prosthetic devices, and are presumably due to changes in the open circuit potential of the cathode and anode as a function of faradaic reactions (Huang et al., 1999; Wei and Grill, 2009). It has also been noted that the observed direct current voltage offset increases in proportion to frequency of stimulation (Franke et al., 2014; Vrabec et al., 2016). Unfortunately, the exact magnitude of the voltage offset created at the working electrode during therapeutic stimulation in the current study is unknown, as a large polarizable Pt/Ir coil was used as the reference electrode during voltage transient analysis instead of a non-polarizable Ag/AgCl or saturated calomel electrode reference. In terms of testing for regulatory approval, the choice of reference electrode was made to better mimic *in vivo* monopolar/unipolar stimulation. The implications of stimulation induced aggregation of charged proteins is an interesting phenomenon that will be explored further in future work, but is outside the scope of the present paper.

DC holding potentials within the maximal allowable limits prior to hydrolysis have previously been demonstrated to attract or repel charged molecules in the realm of neurochemical sensing (Heien et al., 2003; Swamy and Venton, 2007). Cathodic holding potentials have been extensively explored by Wightman, Venton and others to attract neurochemicals of interest or repel unwanted interferents to carbon fiber electrodes during fast-scan cyclic voltammetry (Heien et al., 2003; Swamy and Venton, 2007). As similar holding potentials have already been demonstrated to attract or repel electroactive neurochemicals, it is therefore reasonable to speculate that voltage offsets created during therapeutic neuromodulation pulses may also attract or repel charged biomolecules. Although extensive *in vivo* experiments across numerous animal and human studies—including the studies described here—have shown that the generation of a DC offset at these modest levels causes no clinically relevant tissue damage, this phenomenon may still have implications for healing-in of the electrode or functional behavior of nearby neural circuitry.

### Shannon equation

The Shannon equation (Shannon, 1992) is often referred to when estimating neural stimulation safety limits which relates charge density (*D*) and charge (*Q*) per phase in the following equation:

$$\begin{array}{l} \log\left(D\right)=k-\text{log}\left(Q\right) \end{array}$$

where *k* is between 1.5 and 2.0 and defines the boundary between safe and unsafe levels of stimulation. Just beyond this boundary neural damage has been observed (McCreery et al., 1990) and has been speculated to be a result of toxic products resulting from Pt electrode dissolution (Kumsa et al., 2016). However, because this equation is modeled from “flat” Pt disk electrodes on the surface of the brain, it cannot be directly applied to all electrode materials or all tissue (Cogan et al., 2016). Surface coatings like SIROF increase an electrode's charge injection limit before dissolution occurs, therefore increasing the safe/unsafe boundary estimated by the Shannon equation (Cogan et al., 2016). For a 1 mm diameter electrode without coatings to increase fractal dimensions, the Shannon equation estimates a charge density threshold of ~90 μC/cm^2^. The data presented here show that appreciable dissolution did not occur and no stimulation induced tissue damage occurred with SIROF coated electrodes stimulated at levels well beyond 90 μC/cm^2^ (~600 μC/cm^2^ in PBS/BSA solution and 20–255 μC/cm^2^ *in vivo*).

Another theorized mechanism of neural damage arises from toxic products generated from over excitation of many neurons known as mass action theory (Merrill et al., 2005). While this is possible in high density neural tissue, such as brain tissue, this will likely not occur with stimulation of sparsely distributed baroreceptor nerve fibers located within the carotid sinus. Furthermore, the Shannon equation is modeled from data in which the excitable tissue was directly adjacent to the disk electrode, a scenario categorized by Shannon as a “near-field” case. Given that the baroreceptors are 500 μm or further from the neo electrode, BAT does not fall into the near-field category and the limits proposed by Shannon cannot be applied. Human studies have demonstrated that clinically significant functionality is preserved after many months of BAT with the neo electrode design (Hoppe et al., 2012; Gronda et al., 2014; Abraham et al., 2015).

### Impact of good laboratory practice requirements

The requirement of GLP for critical elements of the safety testing constrains the types of tests available for regulatory approval. GLP requires that “a testing facility shall have a quality assurance unit which shall be responsible for monitoring of each study to assure management that the facilities, equipment, personnel, methods, practices, and controls are in conformance” (FDA, 2016). In addition, the quality assurance unit “shall be entirely separate from and independent of the personnel engaged in direction and conduct of that study” (FDA, 2016). Finally, “a designated representative of the FDA shall have access to written procedures established for the inspection and may request the testing facilities to certify that inspections are being implemented, performed, documented, and followed-up…” (FDA, 2016). GLP is intended to ensure that the manufacturer cannot alter/manipulate data in a study, but also limits the studies to those routinely performed by the GLP-compliant facility. Although it is possible to work with a GLP-compliant facility to develop new tests, the additional cost in money and time for the GLP-compliant facility to demonstrate competency with the new tests is often prohibitive (FDA, 2016). As a result, device manufacturers tend to execute safety testing plans for regulatory approval based off of historical testing plans that have led to regulatory success, and are slow to incorporate newer tests into the process.

### Decision to not evaluate platinum and iridium dissolution *in vivo*

Although the post-mortem animal data did include organ sample procurement to evaluate damage to the liver, kidney, etc. that may have occurred as a function of Pt/Ir toxicity, trace metal analysis of the tissue in the vicinity of the electrode for Pt/Ir material was not performed. In practice, it is functionally problematic to remove all tissue from the surface of a SIROF-coated electrode for analysis without causing delamination of the coating through direct manipulation with surgical tools commonly used for fine dissection of tissue. Given the very real possibility of obtaining flawed data through Pt/Ir damage due to micro-dissection, and the lack of scientific evidence in literature relating direct measurement of any specific Pt/Ir concentrations in the vicinity of the stimulating electrode to clinically significant tissue damage, the decision was made to focus on post-mortem analyses with better established relevance to clinical safety concerns. Subjective microscopic examination of the electrode coating post-mortem at 200× magnification revealed no detectable change in the SIROF coating after 6 months of continuous stimulation.

### Inclusion of additional supporting data in regulatory submissions

The regulatory submissions to both the European notified body and the FDA contained an enormous amount of data. The studies and data presented in this work are by no means all-inclusive of the data submitted to the regulatory bodies. In support of electrical stimulation safety, the regulatory submissions included all prior animal experience with the neo and precursor similar electrode concepts in the canine model taken under both GLP and non-GLP conditions. Although the data described in this paper provided the core foundation for those regulatory submissions, it is difficult to predict if this data set would have been sufficient for regulatory approval in isolation. However, in the experience of the authors the data described in this paper are very similar in extent to other similar FDA regulatory submissions for neuromodulation electrodes.

### Summary of neo human safety data to date

The first-generation CVRx device, Rheos™, consisted of a bilateral implant utilizing a tripolar cuff design similar in size and configuration to common vagal nerve stimulators, but with a proprietary insulating glove with four fingers instead of an insulating wrap. The glove design enabled anchoring the fingers of the glove around branches of the carotid to ensure fixation of the cathode to the carotid sinus bulb. The procedure required an extensive surgical window and a 360° dissection around the carotid artery to wrap the glove design. As a result of bilateral implantation, a large surgical window, and circumferential dissection of the carotid sinus, the rate of freedom from surgical procedure- and system-related complications was 71.3% in the U.S. Pivotal trial through 12 months (data on file, CVRx). In particular, 4.8% of patients received cranial nerve injuries with residual deficits as part of the surgical procedure associated with the dissection around the carotid sinus and implantation of the Rheos electrode (Bisognano et al., 2011).

Two clinical trials have currently implemented the second generation neo system. A nonrandomized study in 30 patients with resistant hypertension demonstrated a rate of freedom from procedure and system-related complications of 86.7% through 6 months (Hoppe et al., 2012). More recently, a randomized study in patients with heart failure exhibited a procedure- and system-related complication event-free rate of 85.9% in 64 patients through 6 months (Abraham et al., 2015). Only one complication was associated with the surgical placement of the electrode which entailed a numbing sensation near the incision due to a transection of a cervical skin nerve (Weaver et al., 2016). All other complications resolved without residual side effects and demonstrated an overall safety profile comparable to that of a cardiac pacemaker (Hoppe et al., 2012).

Of all reported adverse events, none have been associated with electrode dissolution or tissue damage caused as a function of stimulation through the neo electrode-tissue interface. The most typical adverse event related to electrical stimulation of the carotid sinus bulb is referred pain and throat tightening—yet again similar to the vagal nerve stimulation devices—which can be managed by limiting therapeutic settings below the threshold for perceptible sensations. As surgeons gain experience with the Barostim neo implantation procedure in human patients, overall procedure time improves as well as the time to map the neo electrode to minimize referred pain and optimize therapeutic benefit [51]. Further experience with the surgical placement of the neo electrode across a variety of centers is expected to further improve this underlying trend.

### Other considerations and final thoughts

Although the field of bioelectronics medicines was originally dominated by a few select large companies such as Medtronic, St. Jude Medical, Boston Scientific, and LivaNova (formerly Cyberonics) due to the complexity of manufacturing and high cost of pre-clinical and clinical testing necessary for European or United States market approval, smaller companies have increasingly been able to successfully navigate the long-path to market approval, insurance reimbursement and sustainable commercial dissemination. Smaller companies such as Inspire, Second Sight, Enteromedics, StimWave, Nevro, NeuroPace, Spinal Modulation^2^, and CVRx have all obtained a market approval from the Food and Drug Administration (FDA) within the last 5 years, with many more receiving a CE mark allowing sale within the European Union. Moreover, a number of government programs have recently been launched to fund safety testing of new neuromodulation device concepts necessary to enable early feasibility clinical studies, including the NINDS CREATE Devices Program, the NIH BRAIN Translational Programs, and the DARPA HAPTIX, RAM, SUBNETS, and NESD Programs. Many of these new programs encourage the development of high-density or minimally-invasive electrode designs, both of which require miniaturization of the electrode. This, in turn can create additional safety concerns due to the application of greater current densities (Cogan et al., 2016), further motivating the need for rigorous safety testing.

The focus of this paper was to provide the key tests and corresponding summary of results in support of chronic safety for therapeutic electrical stimulation of a SIROF coated, minimally-invasive neuromodulation electrode for both European and United States regulatory submissions. Electrical stimulation safety testing is but one element of the testing necessary to support the overall safety and efficacy of Class III implantable systems for regulatory approval. A non-exhaustive list of other testing requirements includes common tests, such as accelerated flex/fatigue testing of the electrodes following guidelines in section 23.5 of EN45502-2-1, electrical stimulation testing in compliance with ANSI/AAMI ES 60601, and basic biocompatibility testing according to ISO 10993.

The goal of this paper is not to provide a “step-by-step” cookbook for safety testing of a chronically implantable electrode interface for activation of nervous tissue, but to provide a successful starting point as a frame of reference. This paper is also intended to encourage transparency of safety data with an eye toward establishing a large data set across neuromodulation device types, in order to improve the efficiency and predictivity of safety testing across the field. Testing sufficient for regulatory approval is dependent on a variety of factors that are specific to a given neural-interface and therapeutic application that define both benefit and risk. Consequently, the most important recommendation is to establish an ongoing dialogue with regulatory officials as early as possible in the device development process.

## Conclusion

SIROF coatings have been demonstrated in other studies to be suitable for safe stimulation in terms of electrode stability and tissue damage at charge densities well in excess of 1 mC/cm^2^ (Cogan, 2008; Cogan et al., 2009). In this study it was shown that maximum therapy parameters for the neo system correspond to a charge density of ~0.6 mC/cm^2^, which is well within the limits for safe stimulation through SIROF. Voltage transient analysis of stimulation through our SIROF-coated neo electrode also demonstrate that neo stimulation levels are well under reasonable SIROF safety limits (Cogan, 2008). Weekly measurements of electrode stability, including visual inspection using 200× microscopy, EIS, IPG impedance measurements, cyclic voltammetry measurements and ICP-MS trace metal analysis corroborate the voltage transient analysis. *In vivo* chronic GLP safety studies found no clinically significant difference between stimulated electrodes and unstimulated controls at 12 and 24 weeks. These safety data are further strengthened by results in over 90 human patients, with complication-free rates at 6 months comparing favorably to the cardiac pacemaker. All measures within this report demonstrated that the neo electrode is suitable for a lifetime of stimulation at maximum therapy settings without concern for significant electrode deterioration or localized tissue damage. As a result, the neo electrodes were awarded a CE mark for sale in the European Union and are undergoing a U.S. Pivotal trial in the hopes of opening this effective therapy option to U.S. patients.

## Author contributions

SW and SH were primarily responsible for drafting and revising the work, analysing, and interpreting the data. ER contributed data analysis and presentation for the *in vivo* work and EN contributed data analysis and presentation for the benchtop studies. PP and AC assisted in the design and execution of this project. KL was primarily responsible for the conception and design of the work and contributed extensively to data analysis, drafting, and revising.

### Conflict of interest statement

KL and AC are former employees of CVRx who led the efforts to establish the chronic safety of the neo electrode concept for electrical stimulation of the baroreceptors. After leaving CVRx they have maintained no financial ties to the company. SW and PP are paid employees at CVRx. The other authors declare that the research was conducted in the absence of any commercial or financial relationships that could be construed as a potential conflict of interest.

## Footnotes

^1^The FDA Retinal Prosthetic Guidance recommends designing safety studies to support an implanted duration of at least five years. (See https://www.fda.gov/RegulatoryInformation/Guidances/ucm341954.htm).

^2^Spinal Modulation was acquired by St. Jude Medical in 2015.
