# Supplementary material for: Non-clinical and Pre-clinical Testing to Demonstrate Safety of the Barostim Neo Electrode for Activation of Carotid Baroreceptors in Chronic Human Implants
Source: Front Neurosci. 2017 Aug 2;11:438. doi: 10.3389/fnins.2017.00438 (PMC5539240; doi:10.3389/fnins.2017.00438)
Supplement: Supplementary file 1 [file DataSheet1.pdf]

Supplementary Material

Non-clinical and Pre-clinical Testing to Demonstrate Safety of the Barostim Neo Electrode for Activation of Carotid Baroreceptors in Chronic Human Implants

Seth J. Wilks[§], Seth A. Hara[§], Erika K. Ross, Evan N. Nicolai, Paul A. Pignato, Adam W. Cates, and Kip A. Ludwig[*]

*** Correspondence:** Ludwig.kip@mayo.edu

§ These authors contributed equally to the work.

1 Methods

1.1 Long-term Stimulation Testing

As BSA will denature over time, and PBS/BSA will grow bacteria, the PBS/BSA solution was changed every 2 days. Previously used PBS/BSA solution was immediately stored at -4°C and later sent out for analysis of Platinum/Iridium (Pt/Ir) content. Electrodes were not stimulated on weekends or holidays, but instead left in a PBS solution without BSA on those days. A large Pt/Ir coil (exposed surface area greater than 100× the exposed surface area of the neo electrode) was used to ensure minimal charging at the anode, and therefore dissolution of material at the anode should not be considered an issue.

1.2 Weekly Imaging of Electrode Surface

After assembly, baseline 200× images of the coated electrode surface were taken for comparison prior to the application of the stimulation waveform. Inspection of these baseline photos enabled a qualitative assessment of coatings for consistency between electrodes, uniformity of coating over a surface, handling damage, and insulation overlap. Comparing 200× images taken after long-term stimulation *in vitro* to these baseline images enabled qualitative assessment of coating delamination, discoloration of the underlying surface due to undesirable oxidation/reduction reactions, and handling damage to the surface over time. The intensity of the microscope light source was controlled by user manipulation of an unlabeled analog dial (as per most microscopes). Consequently, reflections on the surface of the coating varied from session to session, depending on the unknown intensity of the microscope light source. Beginning week 6, the electrodes were lightly sprayed with deionized water every four weeks to remove adsorbed protein.

1.3 Weekly Electrode Cyclic Voltammetry (CV) Testing

The shaded region in the sputtered iridium oxide film (SIROF) CV in Supplementary Figure 1 represents a cathodic charge storage capacity (CSC_c) of 165.9 mC/cm^2, calculated at a sweep rate of 50 mV/s, noting that the potential axis is related to time through the sweep rate. The CSC_c, which is obtained under low current density, near equilibrium conditions, has limitations as a predictor of safe

charge-injection capacity for neural stimulation. Factors such as pore resistance and activation overpotential, which are most relevant at high current densities and shorter pulse widths typically used for neural stimulation, reduce the charge-injection capacity of an electrode relative to the CSC_c. With typical stimulation parameters, between 5% and 20% of the equilibrium CSC_c can be delivered in a cathodal current pulse without exceeding the water reduction potential (Cogan 2008). For platinum and iridium oxide electrodes, the water window is typically taken as −0.6 V to 0.8 V referenced to a Ag/AgCl electrode. As a saturated calomel electrode (SCE) was used in place of a Ag/AgCl reference for the experiments in the present study, the water window limits were adjusted accordingly to -0.547 to 0.847 V (Cogan 2008). A scan rate of 50 mV/s was used for these measurements.

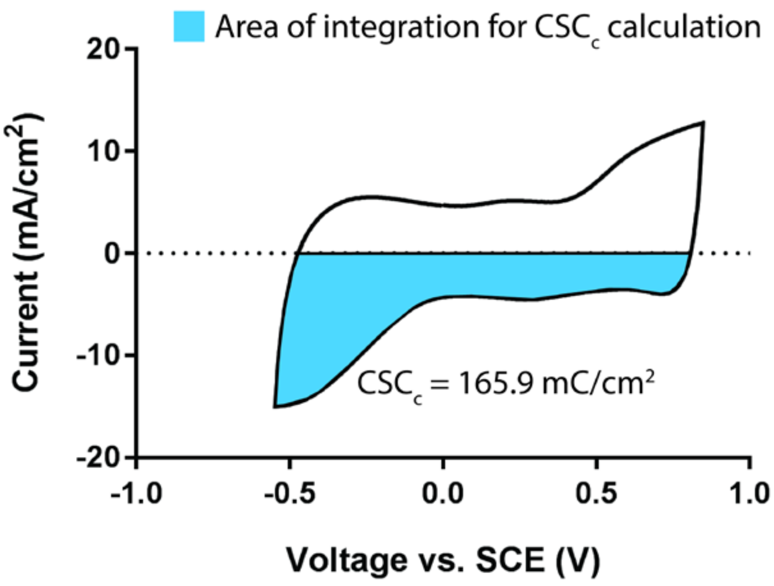

Supplementary Figure 1. Example cyclic voltammetry and CSC_c calculation, as described in (Cogan 2008).

1.4 Periodic Electrode Voltage Transient Measurements

Voltage transient measurements are frequently used to estimate the maximum charge that can be safely injected into tissue during a current-controlled charge balanced stimulation pulse (Cogan 2008). Unlike CV measurements, the stimulation pulse is identical to that used for therapy, and therefore more accurately estimates the safety limits for stimulation. For *in vitro* measurements, the voltage transient is typically recorded in a three-electrode configuration using a large-area return electrode and a non-current carrying reference electrode.

There are several elements that contribute to the voltage transient, and these must be accounted for in the calculation of the maximum catholic polarization potential (E_{mc}). The contributing factors are the ohmic voltage drop V_a ($V_a = i_c \cdot R_i$, R_i = electrolyte resistance and i_c = applied cathodic current) arising from the ionic conductivity of the electrolyte, and the equilibrium potential of the electrode (E_e). As a non-current carrying SCE was not used for this experiment, the equilibrium potential E_e represents the difference in half-cell potentials between the Pt/Ir coil and the neo electrode. Supplementary Figure 2 demonstrates a typical voltage transient, and depicts E_e, along with which

portions of the voltage waveform arise from E_{mc} and V_a. A quick and accurate way to calculate E_{mc} is to subtract the equilibrium potential (E_e) from the residual voltage R_v left on the electrode immediately after the cathodic pulse and immediately prior to the anodic pulse (Cogan 2008). In Supplementary Figure 2, the R_v is 0.6 V and E_e is 0.89 V, meaning that E_{mc} = 0.6 V – 0.89 V, or -0.29 V.

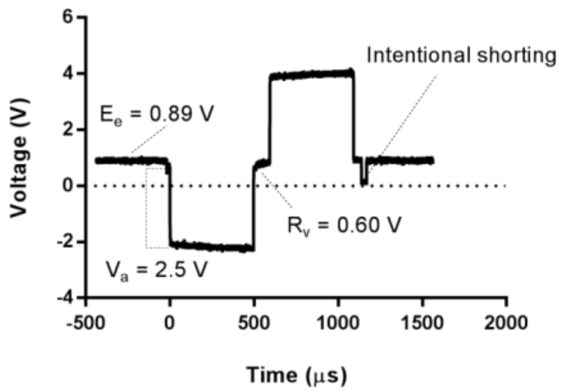

Supplementary Figure 2. Example voltage transient resulting from maximum charge balanced constant current therapy. Note that after every charge balanced pulse, the pulse generator shorts the cathode to the anode for 62.5 µs. In this example, $E_{mc} = R_v - E_e$, or -0.29 V. -0.29 V is well beneath the established safe cathodic limit of -0.6 V. Consequently, stimulating at this level long-term will not cause hydrolysis, and therefore will not cause appreciable tissue damage or dissolution of electrode material.

1.5 Electrochemical Impedance Spectroscopy (EIS) Measurements

EIS measurements taken at the nine week time point for stimulated and unstimulated electrodes in 0.9% NaCl solution were compared to a sample EIS measurement obtained by GreatBatch, Inc. in 0.9% NaCl solution soon after initial sputter coating the Pt/Ir disks with iridium oxide. In-house EIS measurements were made using an Autolab potentiostat PGSTAT300N (Eco Chemie, Utrecht, The Netherlands) with associated frequency response analyzer (Brinkmann, Westbury, NY). A solution of 0.1M PBS (pH 7.4) was used as the electrolyte in a three-electrode cell. A large platinum coil was used as the counter electrode (greater than 100x the surface area of the neo electrode), a saturated calomel electrode (SCE) was used as the reference electrode, and the neo electrode was used as the working electrode. A 10 mV AC sinusoid (peak to peak) waveform was used as the input signal with the DC potential set to 0 V. Impedance values were obtained at ten discrete frequencies per decade spanning 1 Hz to 10 kHz. By measuring impedances over this frequency spectrum, frequency dependent changes in electrode impedances over time could be observed (Cogan 2008). Impedances outside of this frequency band are well outside the range of baroreflex activation therapy stimulation parameters, and therefore provide little relevant information for this application.

1.6 Trace Metals Analysis of Solution Used for Stimulation by Inductively Coupled Plasma Mass Spectroscopy (ICP-MS)

PBS/BSA solutions used for two day sets of stimulation testing were stored in a refrigerator after testing. No samples were taken on week 8 as it was a holiday. Samples underwent ICP-MS testing at Legend Technical Service Inc. to determine levels of platinum and iridium dissolution into solution

during stimulation. Note that some dissolution of material is always expected at any level of stimulation (Robblee, McHardy et al. 1983; Cogan 2008). It is assumed that the total amount of material found in solution during a two day stimulation interval is the total amount of material lost by the electrode during this period. Supplementary Table 1 lists the total amounts of Pt and Ir detected in the solution at the time points of interest.

1.7 Chronic *In Vivo* Study Visual and Electrical Electrode Inspection

The electrodes were dissected free from the carotid arteries, photographed, and visually inspected via light microscopy. There was no evidence of damage (dissolution or degradation) on any of the electrodes and the electrode backers appeared intact. The neo electrode left a faint impression at the implant sites, known as a "witness mark"; however, there were no signs of erosion noted at any implant site. All noted instances of electrode backer damage are attributed to the dissection of the carotid arteries from the animal followed by electrode removal from the fixed carotid arteries.

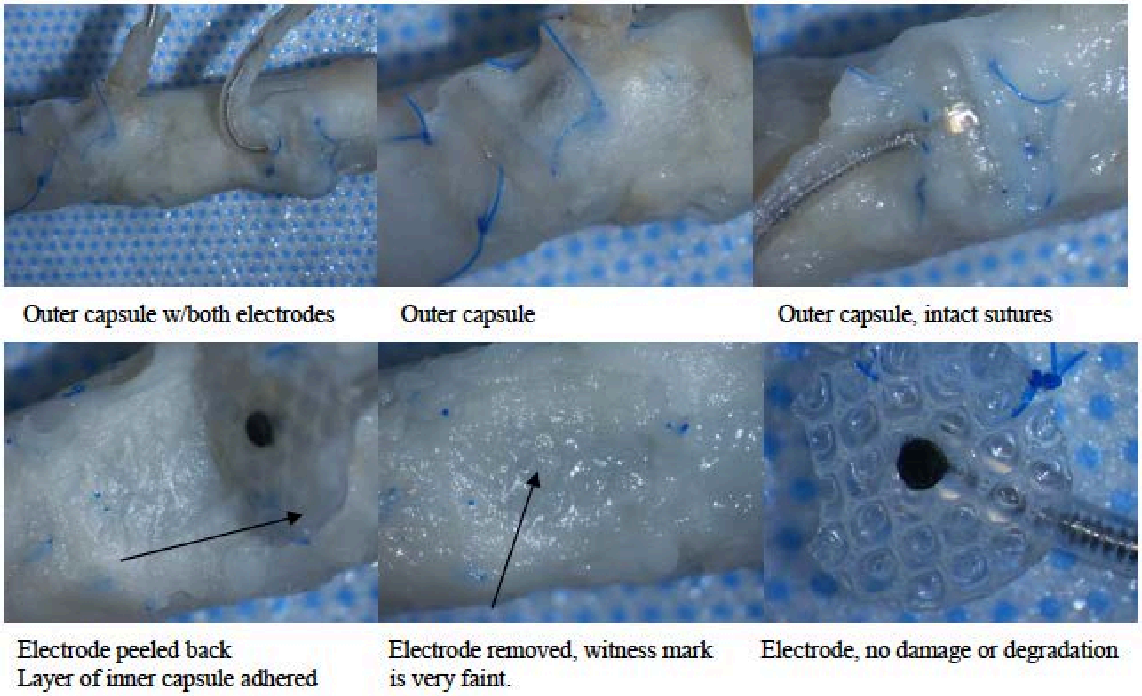

Supplementary Figure 3. Example microdissection of a neo electrode from the carotid artery. The electrode and sutures remained in place and intact and no damage was observed either in the tissue or on the electrode.

Electrical inspection was done by measuring the DC resistance to verify continuity at the electrode to coil joint. A non-contact water bath method was used for all of the electrodes. Using this method, the electrode and probes were immersed in a saline water bath to verify continuity with a digital multi-meter. All were shown to have continuity.

2 Results

2.1 Weekly Imaging of Electrode Surface

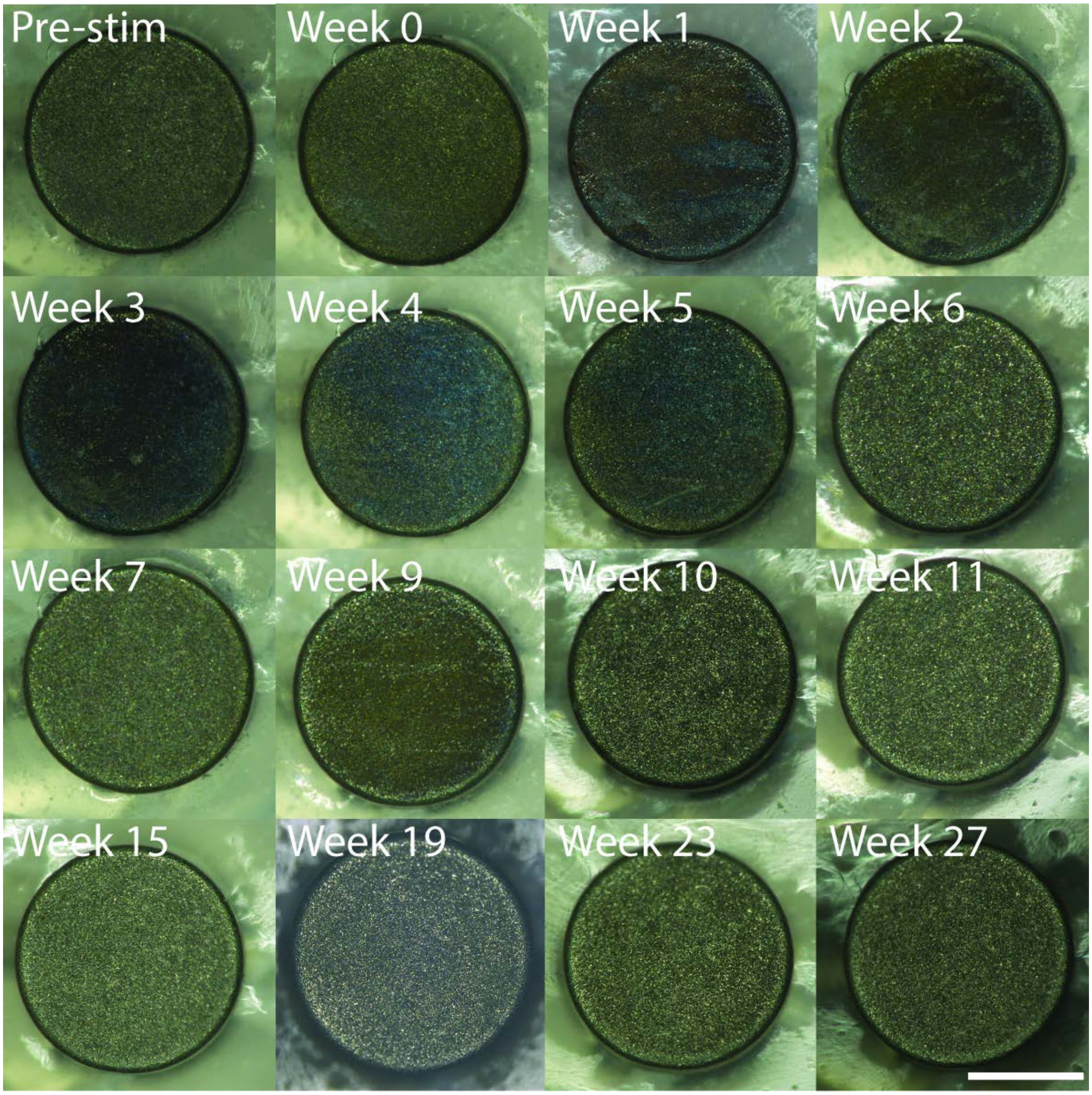

Supplementary Figure 4. 200× optical micrographs of a representative stimulated electrode, Electrode 202, over 27 weeks of stimulation. Scale bar = 0.5 mm.

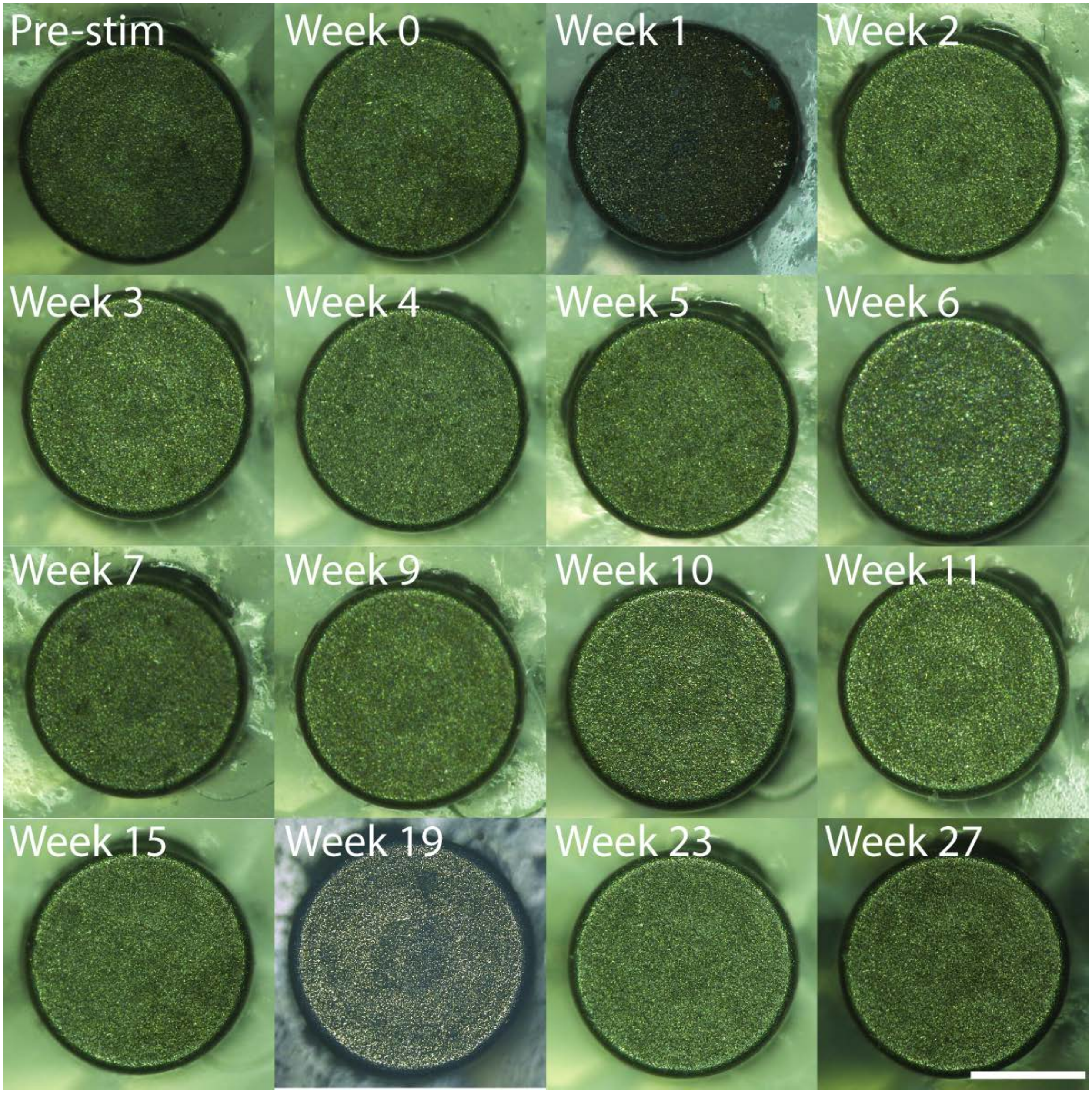

Supplementary Figure 5. 200× optical micrographs of a representative unstimulated electrode, Electrode 205, over 27 weeks of soaking. Scale bar = 0.5 mm.

2.2 Implantable Pulse Generator Impedance Measurements

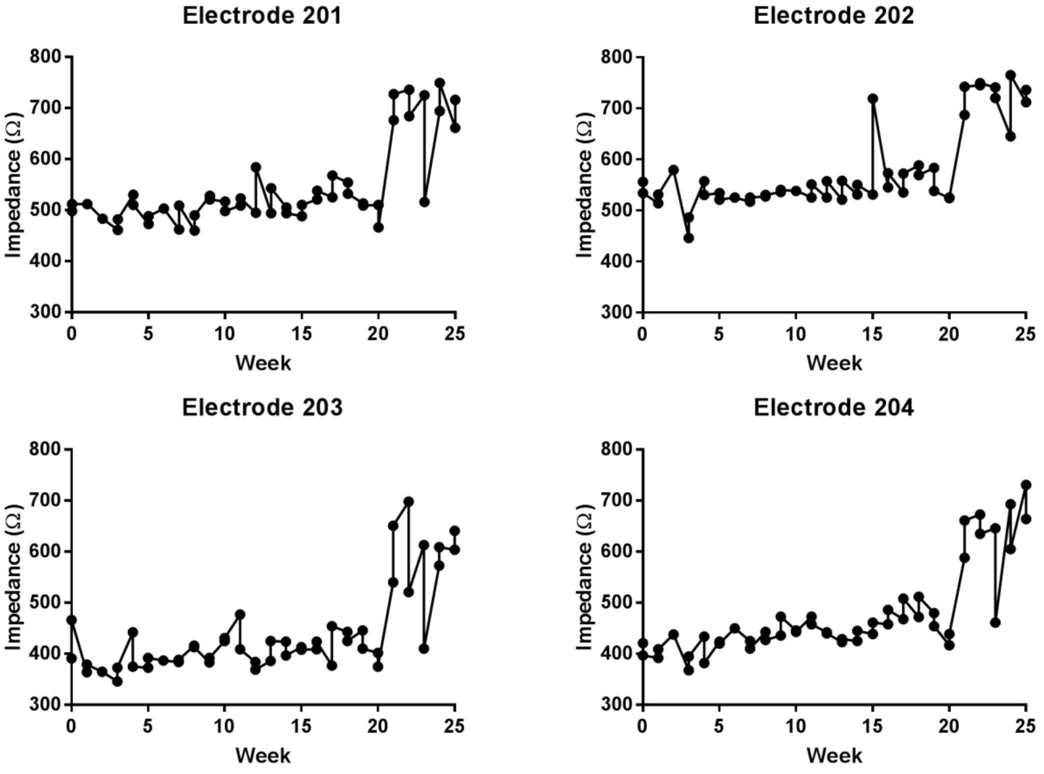

Supplementary Figure 6. Impedances measured by the implantable pulse generator over time. The IPGs was replaced on week 21, resulting in an offset due to intrinsic impedance differences between IPGs. Due to the fact that these impedances were calculated by measuring the voltage at a specific instant in time resulting from a single constant current pulse, slight variability between measurements is expected.

2.3 Weekly Electrode Cyclic Voltammetry (CV) Testing

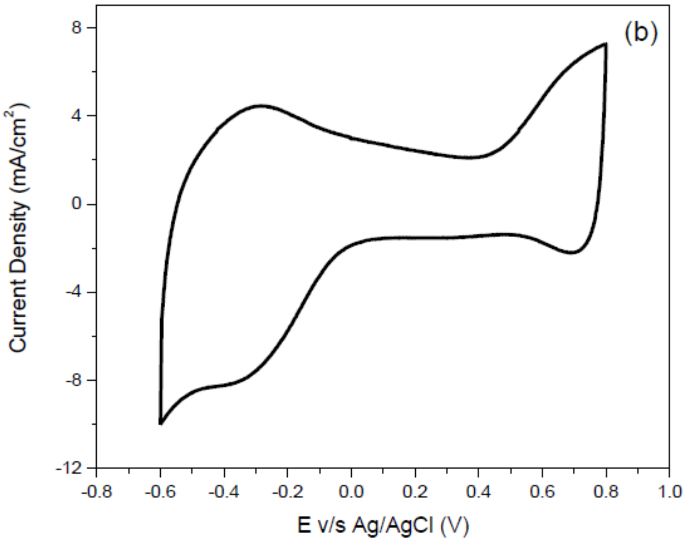

Supplementary Figure 7. CV of SIROF-coated neo electrode taken at GreatBAtch. CSC_c was calculated to be 101.2 mC/cm^2.

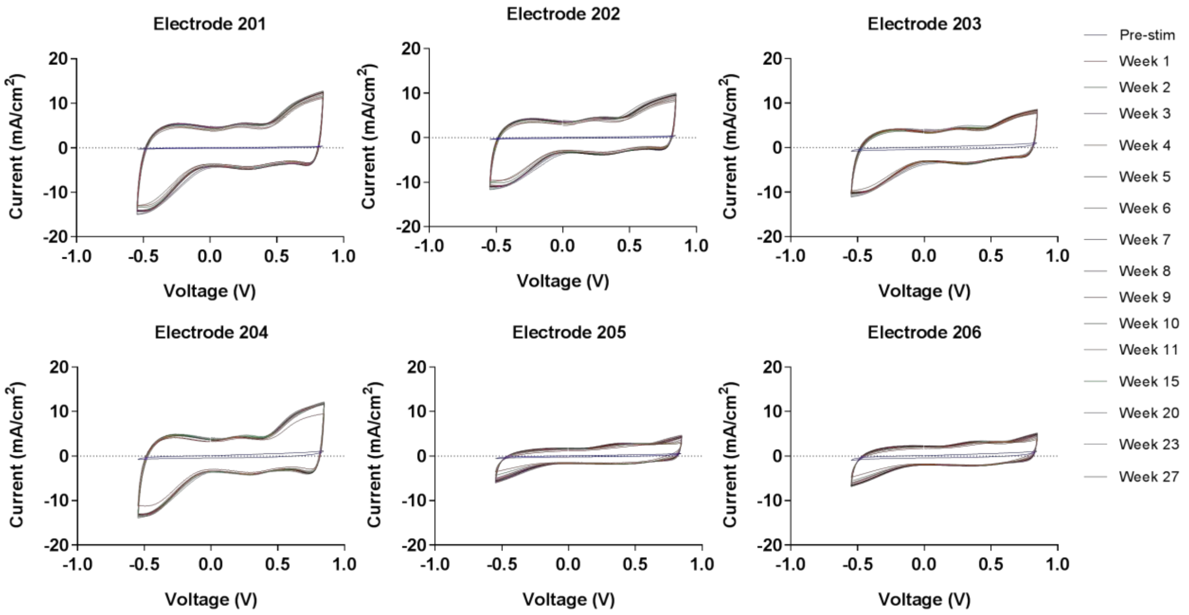

Supplementary Figure 8. Weekly CV measurements of SIROF-coated neo electrodes. Deactivation of the coating due to the unstimulated interval between SIROF application and testing was observed, but full activation was achieved within three weeks for the stimulated electrodes, as expected.

Supplementary Table 1. Measured CSC_c over time. Electrodes 205 and 206 are the unstimulated electrodes. A spreadsheet was used to calculate the area.

	CSC_c (mC/cm^2) by electrode							CSC_c (mC/cm^2) by electrode					
**Week**	**201**	**202**	**203**	**204**	**205**	**206**	**Week**	**201**	**202**	**203**	**204**	**205**	**206**
**Pre-**	2.5	3.2	8.4	8.7	4.2	8.8	**8**	162.3	130.1	118.7	144.4	56.5	69.7
**1**	145.2	112.8	116.7	126.7	40.1	53.7	**9**	163.9	130.1	120.2	144.8	57.8	70.9
**2**	153.8	122.2	118.2	147.6	47.5	61.1	**10**	163.2	130.0	119.2	144.7	57.9	70.8
**3**	161.6	127.8	118.4	146.0	51.1	64.0	**11**	163.9	130.7	121.3	145.8	58.4	71.6
**4**	162.0	128.6	117.5	144.2	52.6	65.2	**15**	161.9	128.9	118.8	144.2	57.2	70.5
**5**	163.4	131.2	119.9	146.5	54.6	68.0	**20**	166.7	132.3	123.4	148.6	57.7	70.1
**6**	141.9	110.9	109.7	140.8	53.6	62.0	**23**	161.2	130.4	120.1	147.3	58.0	70.1
**7**	161.6	129.3	119.3	144.8	55.3	68.1	**27**	165.9	132.0	122.0	149.3	59.3	71.1

2.4 Periodic Electrode Voltage Transient Measurements

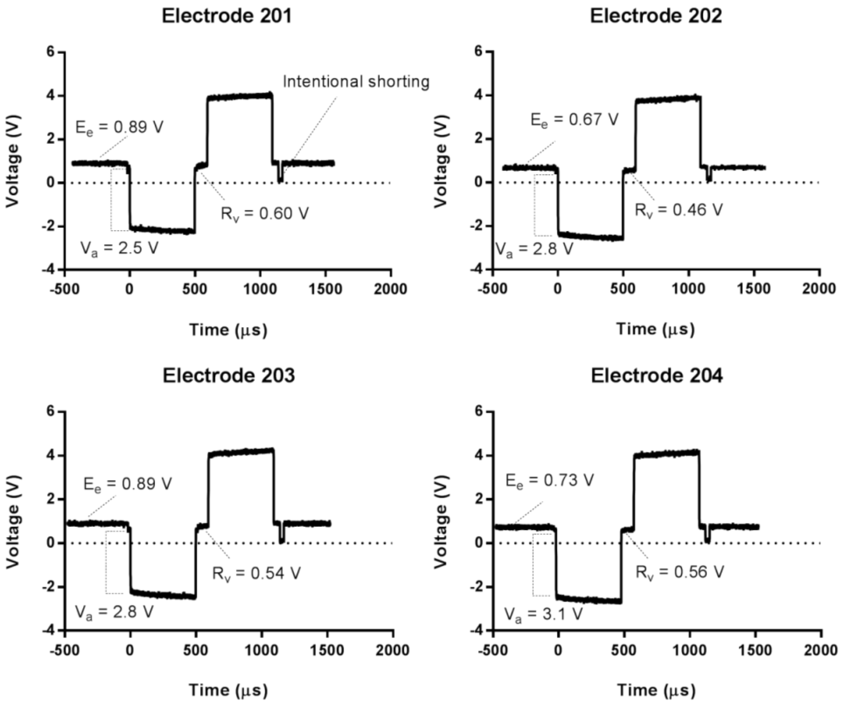

Supplementary Figure 9. Voltage transient resulting from constant current stimulation of Electrodes 201-204 at maximum therapy parameters at Week 6. Note that after every charge-balanced pulse, the pulse generator shorts the cathode to the anode for 62.5 μs.

2.5 Electrochemical Impedance Spectroscopy (EIS) Measurements

After nine weeks of stimulation, phase and impedance magnitude plots over the frequency ranges of 1 Hz to 10 kHz were very similar in form across stimulated and unstimulated electrodes and had changed very little in comparison to impedance spectroscopy measurements taken by the manufacturer soon after application of the SIROF coating to the neo electrodes. Stimulated electrodes demonstrated slightly lower impedance across all frequencies than unstimulated control electrodes due to the expected activation of the hydrous iridium oxide. The phase and impedance magnitude after nine weeks of stimulation were very similar to that measured by the manufacturer soon after coating with SIROF (Supplementary Figure 3). This similarity indicates that any changes to the SIROF coating or the underlying Pt/Ir disk substrate as a result of the electrode-lead body assembly process or electrical stimulation did not compromise the integrity of the electrodes.

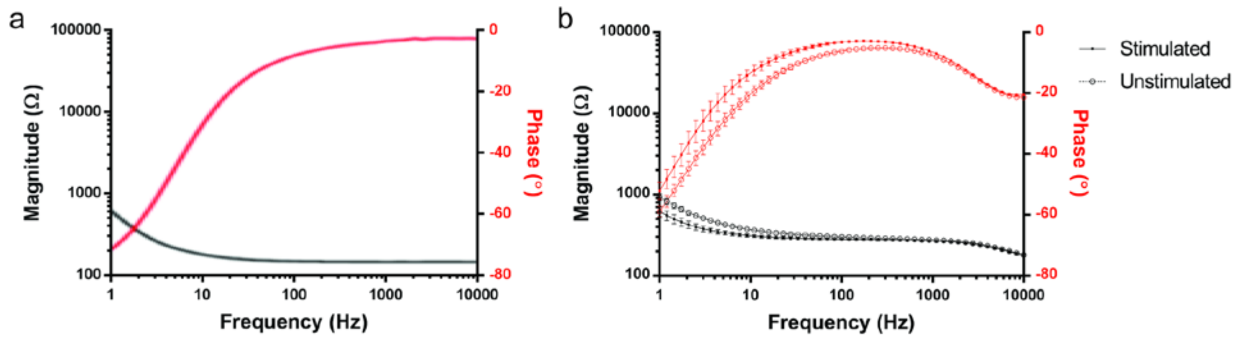

Supplementary Figure 10. EIS immediately after coating and after nine weeks of stimulation. The red line denotes phase and the black line denotes impedance magnitude. The measurements taken by GreatBatch, Inc. immediately after SIROF coating (a) and those taken in-house after nine weeks of stimulation (b) exhibit the same general form. A slight shift in phase and magnitude is expected, as the finished neo assemblies include additional resistance and capacitance added by the cable lead body and terminal pins. Note in Figure 4b that the unstimulated electrodes (open circles) have slightly greater impedance at week 9 than the stimulated electrodes (closed squares).

2.6 Trace Metals Analysis of Solution Used for Stimulation by Inductively Coupled Plasma Mass Spectroscopy (ICP-MS)

Supplementary Table 2. Pt and Ir (in ng) found in solution during two day intervals. An entry of "–" means that the amount of dissolution was below detection limits (12.5 ng). Electrodes 205 and 206 were left unstimulated.

Electrode	201		202		203		204		205		206	
**Material Type**	**Ir**	**Pt**	**Ir**	**Pt**	**Ir**	**Pt**	**Ir**	**Pt**	**Ir**	**P**	**Ir**	**P**
**Week 1**	67.	16	37.5	13	45	21	45	14.2	30	–	37.	–
**2**	17.	19.5	14.7	14	18	21	14.	22.2	–	–	18	–
**3**	21.	50	22	40	17	40	13.	40	–	–	–	–
**4**	17.	16	17	–	–	19.5	–	14.2	13	–	–	–
**5**	–	15.7	16.5	13.2	16.	13.7	16	14	–	–	–	–
**6**	14	17.2	15	14.2	18.	23.7	13.	14	–	–	–	–
**7**	37.	57.5	30	52.5	37.	55	37.	77.5	24.	–	20.	–
**9**	17	–	15.5	–	20	30	14.	–	–	–	12.	–
**11**	110	–	110	–	97.	14.5	95	14.5	90	–	87.	–
**15**	50	–	60	–	60	–	57.	–	52.	–	47.	–
**19**	45	72.5	67.5	72.5	27.	25	42.	35	–	–	–	–
**23**	30	–	23.5	–	21.	–	21.	–	21	–	20.	–

2.7 *In Vivo* Chronic Study Macroscopic Histological Analysis

No electrode damage was seen. Other than the presence of a witness mark from the electrode, no damage to the tissue was observed.

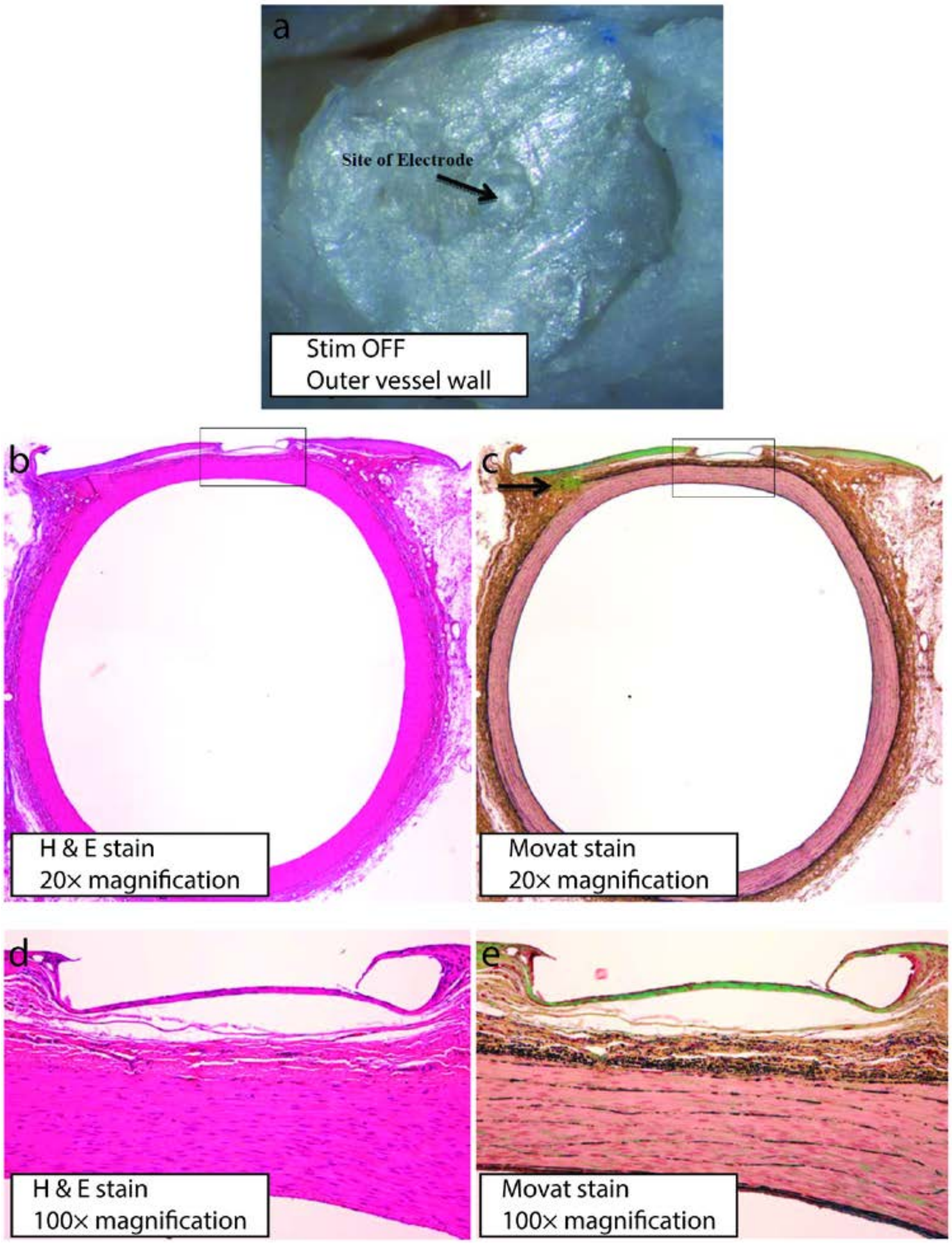

Supplementary Figure 11. Images of the neo implant site (stimulation off) at 24 weeks post-implant. (A) A distinct impression from the electrode was noted on the outer vessel wall following the implant removal at the time of microdissection. Low magnification (20x) H&E stain (B) and Movat staining (C) of carotid artery section showing the thin internal capsule that separated the implant from the underlying vessel wall. H&E stain (D) and Movat staining (E) with higher magnification (100x) of the black rectangular area showing a slight depression and thin internal capsule directly subjacent to the Pt/Ir electrode.

Supplementary Table 3. Microscopic findings at implant site. [1] Implant Site includes proximal backer, step-sectioned electrode block, distal backer. [2] No capsule was present in initial step-section of electrode block. [3] Internal capsule not in close proximity to vessel wall; off to one side.

Animal ID	Implant Site	Presence of Internal Capsule in Sections	Electrode Activity	# Sections Affected/Total Number of Sections at Implant Site[1]		
				Granulomatous Infiltrate of Internal Capsule	MNC Infiltrate of Internal Capsule	Minimal Neointimal Thickening
#10S0019	RC1	Yes	ON	6/10	5/10	9/10
#10S0019	LC2	Yes	ON	13/14[2]	13/14[2]	2/14
#10S0073	RC1	Yes	ON	1/10	0/10	9/10
#10S0073	LC2	Yes	ON	7/10	0/10	9/10
#10S0090	RC2	Yes	ON	8/9	1/9	9/9
#10S0090	LC1	No	ON	NA	NA	6/6
TOTAL				35/53 (66%)	19/53 (36%)	44/53 (83%)
#10S0073	RC2	Yes[3]	OFF	7/11	2/11	6/11
#10S0019	RC2	No	OFF	NA	NA	16/17
#10S0019	LC1	No	OFF	NA	NA	2/16
#10S0073	LC1	No	OFF	NA	NA	10/10
#10S0090	RC1	Yes	OFF	2/6	1/6	0/6
#10S0090	LC2	Yes	OFF	9/9	5/9	9/9
TOTAL				18/26 (69%)	8/26 (31%)	43/69 (62%)

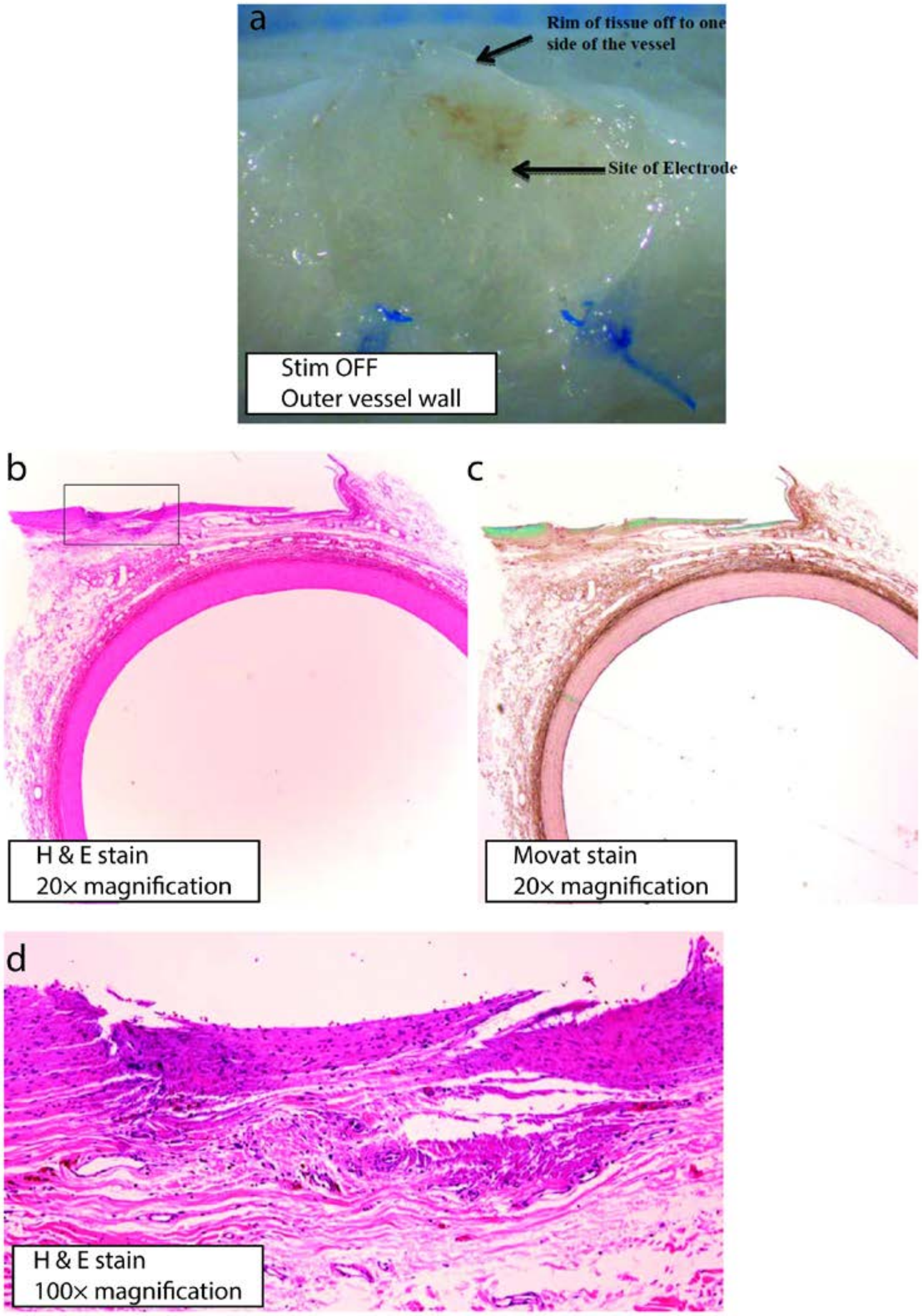

Supplementary Figure 12. Representative image from electrode not apposed to target tissue (A) An indistinct impression from the electrode was noted on the outer vessel wall following implant removal at the time of microdissection. The implant was not closely apposed to the outer wall of the common carotid artery and was off to one side of the vessel. Low magnification (20x) H&E stain (B) and Movat's staining (C) showing the thin internal capsule that separated the implant from the underlying vessel wall. (D) Higher magnification (100x) of the black rectangular area showing the fragmentation and coagulated appearance of the internal capsule subjacent to the electrode.

3 References

Cogan, S. F. (2008). "Neural stimulation and recording electrodes." Annu. Rev. Biomed. Eng. **10**: 275-309.

Robblee, L., J. McHardy, et al. (1983). "Electrical stimulation with Pt electrodes. VII. Dissolution of Pt electrodes during electrical stimulation of the cat cerebral cortex." Journal of neuroscience methods **9**(4): 301-308.
